# Measuring child and adolescent well-being in Denmark: Validation and norming of the Danish KIDSCREEN-10 child/adolescent version in a national representative sample of school pupils in grades five through eight

**DOI:** 10.1371/journal.pone.0291420

**Published:** 2023-09-08

**Authors:** Tine Nielsen, Maiken Pontoppidan, Morten Pettersson, Christina H. Donstrup, Svend Kreiner, Signe Boe Rayce

**Affiliations:** 1 Department of Applied Research in Education and Social Sciences, UCL University College, Odense, Denmark; 2 VIVE Health, VIVE—The Danish Center for Social Science Research, Copenhagen, Denmark; 3 Biostatistics Unit, Department of Public Health, University of Copenhagen, Copenhagen, Denmark; Albanian University, ALBANIA

## Abstract

KIDSCREEN-10 is a generic instrument for measuring global health-related quality of life among 8-18-year-old children and adolescents. This study examines the criterion-related construct validity and psychometric properties of the Danish language version of the KIDSCREEN-10 using Rasch models. A further aim was to construct Danish norms based on the resulting person parameter estimates from the Rasch models. Data consists of a nationally representative cross-sectional survey of 8171 children in the 5^th^ to 8^th^ grade of primary school in Denmark. No adequate fit to the Rasch model or a graphical loglinear Rasch model could be established for the KIDSCREEN-10 in the full sample of children (n = 8171). Results based on analyses with increasing samples sizes showed that even with the smallest sample item 3 (Kid3) of the KIDSCREEN-10 did not fit the Rasch model. After elimination of Kid3, substantial local dependence and differential item functioning relative to gender and grade level was still present. Already with a sample size of 630 fit to the Rasch model or a graphical loglinear Rasch model adjusting for local dependence and differential item functioning was not established. Therefore, generation of Danish norms was not realizable, as this requires valid sum scores and estimates of the person parameters for an adequate number of cases. Thus, the Danish language version of the child/adolescent self-report KIDSCREEN-10 questionnaire cannot be recommended for use in population-level studies. Neither can use in small sample be recommended as adjustment for differential item functioning and local dependence is ambiguous.

## Introduction

Adolescence is characterized by rapid physical, psychological and social changes as well as increased autonomy [1]. Positive well-being and a healthy psychosocial development are pivotal at this stage of life and is associated with higher life satisfaction, learning and less risk behaviours [2–4]. Mental health in adolescence even has consequences beyond adolescence [5, 6] with mental health problems tracking into young adulthood and may negatively impact physical, mental, social, and educational trajectories in a lifetime perspective [6–12]. Adolescent mental health and well-being has gained increasingly attention in Western and European high-income countries [13] as studies indicate declining trends in well-being [13, 14]. Globally, mental health conditions account for 16% of the burden of disease and injury in adolescents and half of all mental health disorders start in adolescence [15]. Consequently, child and adolescent well-being has become highly prioritized at the global level [15, 16]. Adolescence has been highlighted as a critical stage of life in terms of prevention of problems but also as a period with great potential for health promotion [10]. However, to effectively 1) monitor and promote well-being at an overall level, 2) identify adolescents with low level of mental well-being and 3) develop and offer effective interventions to those in need, it is imperative to have valid and accurate measure of well-being among adolescents.

Neither mental health nor well-being are clearly defined concepts, which are regularly used interchangeably [17]. While in the last two decades, it has been increasingly acknowledged that child and adolescent well-being is more than the absence of problems or disease [2, 3, 17–19] and that a high level of well-being may even serve as a buffer against stressful life events [2, 6, 20], this is also subject to academic debate [17, 21]. According to [21] the debate is centred around WHO’s proposed definition of mental health as: *“A state of well-being in which the individual realizes his or her own abilities*, *can cope with the normal stresses of life*, *can work productively and fruitfully*, *and is able to make a contribution to his or her community”* [22], and is concerned with the normative criteria used to define the biomedical and cultural aspects of mental health, the emphasis on positive aspects, and finally the overlap with well-being [21]. The debate includes issues such as whether high mental health/wellbeing and mental illness are two extremes on a mental health continuum or whether a person with mental illness can have good mental health/mental wellbeing [17]. Several scholars have addressed the importance of positive mental health such as high mental well-being, flourishing and high quality of life with key aspects being education, autonomy, competence and social relatedness, and emotional well-being [11, 23, 24]. Therefore, it is imperative with measurement tools, which addresses well-being directly, in order to successfully assess these positive aspects of mental health.

KIDSCREEN [25] is a generic instrument for measuring health-related quality of life (HRQoL) among 8-18-year-old children and adolescents, developed by a group of European experts in child and adolescent health and well-being. KIDSCREEN is highly used in both research and practice in Europe to measure well-being in children and adolescents. Three versions with varying numbers of items exist: KIDSCREEN-52, KIDSCREEN-27 and the KIDSCREEN-10 index. Each version is available in both a child/adolescent-reported and parent-reported version [26]. While the originally developed KIDSCREEN-52, as well as the derived KIDSCREEN-27, are multidimensional measures comprising respectively ten and five dimensions, of HRQoL represented by subscales, the KIDCREEN-10 was developed to measure global HRQoL. Thus, it comprises items from each of the five subscales of the KIDSCREEN-27 [26]. The 10-item index takes only a few minutes to complete and has been recommended for screening purposes by the International Consortium for Health Outcomes Measurement (https://www.kidscreen.org/english/project/) and for large-scale population studies, which typically call for short scales to avoid responder fatigue. The short KIDSCREEN-10 index has been translated into more than 40 languages including Danish. However, although being recommended by the National Board of Social Services in Denmark, the Danish version has never been validated and no Danish norms exists.

The KIDSCREEN-52 questionnaires were developed using Rasch measurement models, however, only by assessment of item fit and presence of differential item functioning (DIF) and not other properties of the Rasch model [27]. Furthermore, item fit was assessed using unconditional infit indices and a rule-of-thump evaluation of fit based on cut-point values, rather than testing item fit. DIF was assessed by a logistic regression method, entailing dichotomization of the items. Results were reported for the reduced 27-item and 10 item versions in the development study as well. For the 10-item child/adolescent questionnaire, item fits were reported as within the chosen interval. DIF results were reported only as min-max intervals for the set of 10 items across countries, gender and age groups [27], and not at the item level. In the KIDSCREEN handbook [27], it was concluded that most items displayed negligible DIF, and a few items displayed sizeable DIF. The developers further proposed that the sizeable DIF could theoretically be attributed to the fact that these items measure secondary aspects which are relevant for HRQoL, but vary across the groups to be compared. Though it was not reported which of the 10 items that displayed DIF, it was evidently DIF across countries [27]. Thus, detailed results at the item level might have provided researchers and users with valuable knowledge on which items has cross-country issues, and DIF analyses within single countries might have been even more informative to researchers.

Criterion, convergent and discriminative validity has subsequently been supported in 13 European language versions of the KIDSCREEN-10 as well as a Japanese language version [28–30]. However, while the psychometric properties of the KIDSCREEN-52 and KIDSCREEN-27 have been examined in several studies, including studies using Rasch models (RM) [31], only four studies [28, 32–34] have examined the psychometric properties and fit to the Rasch model of the KIDSCREEN-10 following its development.

Across studies of adolescent samples from more than 15 countries, the reliability (reported as Cronbach’s Alpha) of the KIDSCREEN-10 ranges between 0.75 and 0.83 [28, 29, 35–38]. Of these, only one study estimated the reliability while taking into account that some of the items in KIDSCREEN-10 were locally dependent [28], who reported Cronbach’s Alpha as 0.79 for the German language version. This suggests that the reliability in some of the other studies may have been inflated.

It is imperative that an assessment measure works equally well independent of participant’s group membership (e.g., age group, gender, race). Adolescence is characterized by many physical and psychological changes, which typically occur at slightly different ages for boys and girls [1]. It is also likely that boys and girls experience e.g., social dynamics differently [39]. Therefore, it is possible that some items will function differently according to age and/or gender. If so, the KIDSCREEN-10 score will be biased to some degree. Müller & Hoti [28] found DIF relative to gender, while Gong et al. [33] found DIF relative to age. Erhart et al. [32] did not test for DIF and Velez et al. [34] did not report evidence of DIF related to gender. Other studies using the KIDSCREEN-10 have found differences between boys and girls showing that especially teenage girls report lower HRQoL than boys [40–43], as well as differences in age showing that HRQoL decreases with age [41–44]. However, it is not possible to know whether and to which degree the observed gender differences in HRQoL are affected by DIF. Adolescent well-being may also be influenced by migration status. An Icelandic study found that support from friends and family was associated with greater adolescent well-being irrespective of migration status, but also that adolescents of foreign origin benefitted more from supportive parents than native-origin adolescents [45]. To our knowledge, only one previous study [28] has examined DIF and overall measurement invariance in relation to migration status within a country. This study found DIF in relation to citizenship status. Thus, there is a need for further investigations into this issue.

The increasing emphasis on the well-being of Danish children and adolescents highlights the necessity for valid measurement tools and addressing any issues related to DIF or overall invariance across subgroups with the KIDSCREEN-10 in Denmark. However, as KIDSCREEN-10 is recommended for use in evaluations of social interventions involving vulnerable children/adolescents, there is also a pressing need for Danish norm data. Such norms can be used to identify children at risk for low well-being and will allow comparisons between vulnerable children/adolescents and the general population of Danish children/adolescents in evaluations.

Currently, the only comparison reference for well-being assessments with the KIDSCREEN questionnaires consists of norms that: 1) were developed in other cultures than the Danish, which is culturally inappropriate [46], and 2) only exists for age groups with a span of 3–4 years, which is usually not considered appropriate for children/adolescents, as their development is progressing at a rapid pace [47]. Additionally, the existing norms are generally too old by European standards for psychological and educational tests and measurement scales [48], with the exception of the newly developed German norms [49].

Therefore, the overall purpose of the present study was to investigate the psychometric properties of the Danish translation of the KIDSCREEN-10 in a population-representative sample of 8171 adolescents attending public school years five through eight (age range 9–15 year-olds), using Rasch models, as in the original development of the KIDSCREEN questionnaires. The aim of the study is twofold: First, to investigate the criterion-related construct validity of the Danish language version of the KIDSCREEN-10 questionnaire as well as its psychometric properties using Rasch models with emphasis on testing for differential item functioning as well as local independence of items. Second, to construct Danish norms based on the resulting person parameter estimates from the Rasch models in order to obtain suitable norms for the Danish context.

The study will be the first validity study of the Danish language child/adolescent version of KIDSCREEN-10, and of any Danish KIDSCREEN questionnaire.

## Methods

### Instrument

KIDSCREEN is a collection of questionnaires for the assessment of the health-related quality of life among children and adolescents (target age range 8 to 18 years), which is widely used to measure well-being, especially in Europe. A European consortium of child and adolescent well-being experts and researchers from 13 countries (*not* including Denmark) developed the KIDSCREEN questionnaires in 2001. The purpose was to develop an instrument to identify (screen) children and adolescents at risk and to monitor child and adolescent well-being. KIDSCREEN is now available in 51 different languages including Danish. KIDSCREEN consists of either 52, 27 or 10 items with the shorter versions included in the longer ones. Thus, the 10-item and the 27-item versions both contain items from all dimensions of the 52-item version except for the bullying dimension. Each version exists in a children/adolescents-reported version and a parent-reported version [25].

In the 10-item version, the children/adolescents are asked to think about the last week, when responding. Two response scales are used: for items 1 and 9 the response scale is “not at all, slightly, moderately, very and extremely” scored 1 to 5. For the remaining items the response scales is “never, seldom, quite often, very often, always” scored 1 to 5. Items 3 and 4 are reversed before adding items to a sum score. Higher scores on the KIDSCREEN-10 indicate a higher degree of well-being.

The KIDSCREEN-10, which we are concerned with in this study, only takes a few minutes to complete. This feature potentially enhances versatility, relevant to both screening and large-scale population studies. These typically require shorter scales to prevent responder fatigue and to maintain cost efficiency. The KIDSCREEN-10 is recommended for screening by the International Consortium for Health Outcomes Measurement (ICHOM; https://www.kidscreen.org/english/project/).

The KIDSCREEN questionnaires were developed using Rasch measurement models, and country-specific norms were developed. However, these norms are now outdated based on test standards, since the used data was from before 2006 [48], and it is not advised to use norms developed for other cultures [46]. While Denmark was not initially involved in developing KIDSCREEN, an official Danish translation is now available (https://www.kidscreen.org/english/language-versions/denmark/). Nevertheless, there are no available norms for Denmark. Despite the lack of validation studies of the Danish version and the lack of Danish norms, the KIDSCREEN questionnaires are on the list of recommended measures from the Danish National Board of Social Services [50] and is being used for various purposes, including research and in relation to family interventions.

The development and initial validation of the original full 52-item version of KIDSCREEN is documented in the KIDSCREEN handbook [27], while the methods used to reduce this to first the 27-item and then the 10-item versions is only described in general terms in the handbook. A later study provided more details for the construction of 27-item version [51], however, details of the reduction to the 10-item version, which is the object of this study, remains unavailable (Personal correspondence with Professor Erhardt on the development and validation of KIDSCREEN-10 revealed that the article [52] referenced in other work was never published).

Since development, the KIDSCREEN questionnaires have been employed in more than a thousand studies worldwide, making it one of the most widely used instruments for the assessment of child/adolescent well-being and HRQol.

### Previous construct validity studies

**Fig 1:**
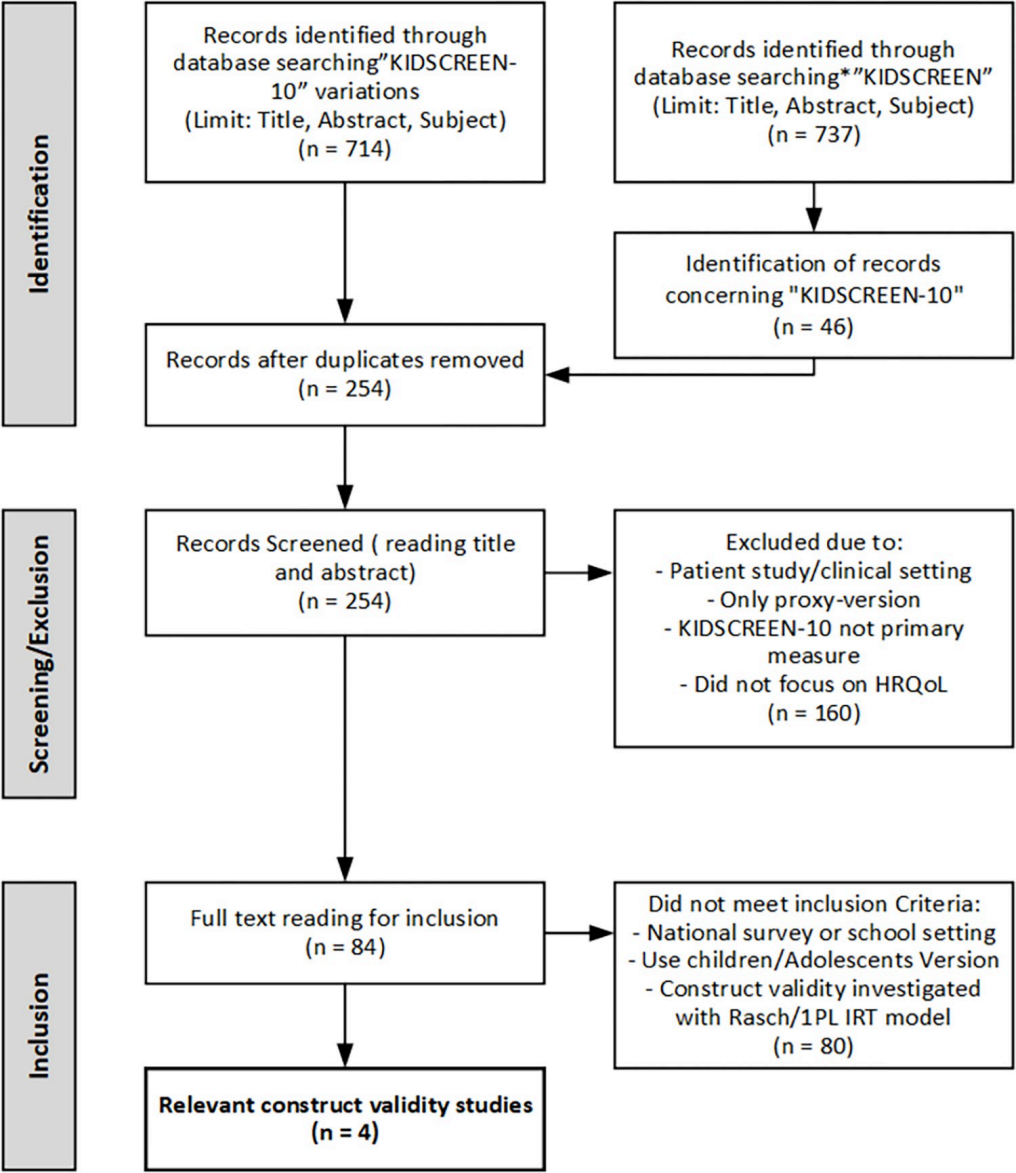
Flow of literature search and results.

In order to locate KIDSCREEN-10 construct validity studies employing the Rasch (or one parameter logistic) model, which was also used in the initial development of the KIDSCREEN questionnaires, we conducted a systematic search for literature in 10 databases (*EMBASE*, *PubMed*, *CINAHL*, *Cochrane*, *ERIC*, *APA PsycInfo*, *Academic Search Premier*, *Scopus*, *Sociological Abstract*, *SocINDEX*). As the term KIDSCREEN-10 is not consistently used in the literature, we used four different search terms; KIDSCREEN-10, KIDSCREEN 10, KIDSCREEN10 and even KIDSCREEN, and searched for these in title, abstract and subject. The search was completed in April 2022, and the evaluation of literature in October 2022. Fig 1 shows the flow and results of the literature search.

The four identified construct validity studies [28, 33, 34, 43] used different language versions of the KIDSCREEN-10 in different countries and tested fit to the Rasch model with varying degrees of rigour.

The first study was Erhart et al. [43], which revisited the psychometric properties of the KIDSCREEN-10 used in various language versions with school children from 15 European countries (n = 78,383, analysed country-wise). Fit to the Rasch model was, however, assessed solely by item fit using (presumable unconditional) infits and outfits and set cut-points for fit (i.e., 0.7–1.3). For 12 of the 15 countries, item Kid9 did not fit the Rasch model (the item fitted only for Greenland, Russia and Macedonia), while item Kid3 did not fit for the Russian sample, according to the criteria set for fit. In addition, the analyses, did not include tests for DIF, local independence of items or overall fit to the Rasch model.

The second study [34], used the Spanish language version of the KIDSCREEN-10 in a Columbian sample of children (n = 416, mean age 11.7 years). Fit to the Rasch model was assessed by unconditional infits and outfits using set cut-points (0.7–1.3), as well as tests for DIF. No issues with item fit were reported, but DIF was found for items Kid3 and Kid4 in relation to socioeconomic status. Local independence of items and overall fit was not assessed.

A third study [33] studied a Chinese language version of the KIDSCREEN-10 with a sample of 1,830 8-18-year-old children and adolescents. This study assessed item fit by unconditional infits and outfits using set cut-points (0.7–1.3) and three items were found misfit to the Rasch model (items Kid3, Kid4 and Kid5). They found DIF relative to age for items Kid3 and Kid4 suggesting that these two items function differently among children and adolescents. After elimination of items Kid3, Kid4 and Kid5, and collapsing two response categories into one, fit to the Rasch model (based on item fit and no DIF) was found for the remaining set of seven items. However, local independence of items or overall fit was not assessed.

The fourth study [28] has provided the most thorough psychometric assessment of the KIDSCREEN-10 questionnaire to date using Rasch models. This study examined the German language version with a sample of 1100 Swiss sixth graders, using both Rasch and graphical loglinear Rasch models. Müller and Hoti (2020) did not find a fit to the pure RM. However, when taking into account local dependence between four item pairs and DIF relative to gender (item Kid10: *Have you been able to pay attention*?) and citizenship (item Kid4: *Have you felt lonely*?*)* into account, responses almost fitted a GLLRM. Thus, there was no evidence against item fit assessed using conditional infits and outfits as well as item-rescore correlations. Also, no evidence against overall homogeneity was found. However, some evidence against overall invariance across gender and citizenship was found, suggesting that there might still be some undiscovered DIF. The findings of Müller and Hoti [28] suggest that KIDSCREEN-10 can potentially provide essentially valid measurement, if LD and DIF are taken into account for.

### Data collection and sample

The data for the current study consists of a nationally representative cross-sectional survey of 8171 children in the 5^th^ to 8^th^ grade of primary school in Denmark. The data was suitable for the current study in two ways. Firstly, the data were useful for evaluating the measurement properties of KIDSCREEN-10 in large-scale population surveys because of the extensive sample and subsamples. Secondly, the data could be used to create norms due to the standardized data collection procedure, representative sample, and sampling methods employed [48]. The data was originally collected as part of the Danish national assessment *Children and young people’s reading 2021*—a project that assesses children’s reading experiences every four years [53]. This project collected random samples from four target populations in the Danish primary school: children in the 5^th^, 6^th^, 7^th^ and 8^th^ grade. The approximate age of children at the different grade levels: 5^th^ grade: 10–11 years old, 6^th^ grade: 11–12 years old, 7^th^ grade: 12–13 years old, 8^th^ grade: 14–15 years old. The data collection took place from September 15^th^ to November 17^th^, 2021. Each student completed an online questionnaire about reading, which also included the KIDSCREEN-10 questions.

The samples were designed as two-stage cluster samples. At stage one, a sample of schools was drawn, and at stage two one intact class of students from each target grade was selected within each school. Schools were either sampled to participate with a class in the 5^th^ and the 6^th^ grade, or with a class in the 7^th^ and the 8^th^ grade. In the first stage, schools were randomly selected with a probability proportional to their size (i.e. number of students). 154 schools were sampled to participate with a class in the 5^th^ and the 6^th^ grade from a list of schools with students enrolled in those target grades. Similarly, 151 schools were sampled to participate with a class in the 7^th^ and 8^th^ grade from another list of schools with students enrolled in these grades. Each list was explicitly stratified according to school type (public or private) to ensure proportional representation and to improve sampling precision. In the second sampling stage, one intact class of students was randomly selected with equal probability from each target grade within each selected school. A selection of classes with equal probability, combined with PPS sampling for schools, should in principle result in a self-weighting student sample [54].

Sample sizes were chosen to ensure that specific precision requirements were met in order to be able to infer precisely to each target grade population in the reading project. Sample estimates of any student-level percentage estimate, in each target grade, should have a confidence interval of ± 3.5 percentage points. Prior to data collection, calculations showed precision requirements would be met with a school sample of 120 and a student sample of around 2000 students in each target grade. To account for potential non-response at the school- and student-level, school sample sizes were increased to the previously mentioned 154 schools (5^th^ and 6^th^ grade) and 151 schools (7^th^ and 8^th^ grade). Further details of the sampling design, coverage and exclusion, data collection, etc. are included as S1 File.

To ensure representativeness in a study, minimizing non-response bias is essential. The participation rates achieved were noteworthy: 70–75% for schools and around 85% for students in each target grade (see S1 File, S1 to S4 Tables). Notably, the sample of students in each target grade were demographically similar to the national population they were supposed to represent (see S1 File, S1-7 to S1-10 Tables for details). This similarity also enhances our confidence in the study’s ability to accurately reflect the larger population of students in the 5^th^ to 8^th^ grade, thus making the data appropriate for generating norms.

The pooled data sample for the current study consists of the KIDSCREEN-10 items and a number of variables with relevant background questions intended for analyses of differential item functioning. Student distributions on these background variables as well as the corresponding population distributions are shown in Table 1. The distribution across grades was approximately equal-sized groups with about 2000 students at each grade level. There was a 50/50 split on boys and girls. The majority of the children attended public school (78.8%), In addition to these register-based information, students were asked about the language spoken in their home; the majority reported this to be Danish (75.0%). We were not able to obtain information on this in the population. In relation to the register-based background information, the difference between sample and population varied between only 0.8 and 1.6 percentage points. We are thus confident that the results of our analyses can be generalized to the population level with regard to Danish students in grades 5 to 8 (i.e. ages approximately 10–15 years).

**Table 1:** Student characteristics compared to population distributions.

Subgroups	Study sample % (n)	Total population %
Gender		
Girl	50.1 (4094)	49.3
Boy	49.9 (4077)	50.7
Grade		
5^th^	25.9 (2119)	24.6
6^th^	25.9 (2118)	25.4
7^th^	24.2 (1976)	24.9
8^th^	24.0 (1958)	25.1
School type		
Public	78.8 (6438)	80.4
Private	21.2 (1733)	19.6
Language spoken in the home		
Danish	75.0 (6127)	*NA*
Other	25.0 (2044)	*NA*
Total	100.0 (8171)	100.0

*Notes*. Information about the population is based on data about all Danish students from the 5^th^ to the 8^th^ grade in the school year 2020/2021. It was collected from the Danish educational data warehouse [Uddannelsesstatistik] available from the Danish Ministry of children and education at https://uddannelsesstatistik.dk/Pages/main.aspx. Data from the sample is based on unweighted data.

#### Ethics

Informed consent was deemed not to be required by UCL University College (the institution of the responsible researchers) prior to the data collection for the original study, as the data was collected strictly for research purposes. This decision was supported by a legal basis, found within the European General Data Protection Regulation (GDPR) Article 6, 1 e (public interest), and §10 in the Danish Data Protection regulation (research purposes).

To ensure compliance with GDPR Article 13, UCL University College provided detailed information to the parents of children selected for the study before the initiation of the data collection. This information included the voluntary nature of participation, an explanation of how personal data would be processed, and an assurance that participants could opt out of the project at any time–both before and after the study. 62 children were excluded from the study based on their own or parents’ wishes.

The study fell within the legal boundaries defined by Danish law, wherein register- and survey-based studies do not require explicit approval from the National Committee for Health Research Ethics. This exemption is specified in part 4, section 14(2) of the Danish Act on Research Ethics Review of Health Research Projects (Legal Information, 2017).

The responsible researchers for the data collection have provided the following details about the information supplied to both schools and parents of children: During the original study, schools received written information indicating their random selection for participation, specifying certain grades, and explaining that a random draw would determine the specific classes involved. The provided information also outlined the study’s aim and purpose, emphasizing the voluntary nature of school participation. In the selected classes, parents were notified (before the study was conducted) in writing about the study, its objectives (i.e., examining reading experiences and well-being, among other things), the conducting researchers, and the voluntary nature of survey participation. They were informed that they could choose to opt-out by notifying the school. Furthermore, parents were informed that their child’s name and school email would be collected solely to provide unique survey links. Additionally, information on gender and birthdate would be collected for research purposes, and they were granted the right to request data deletion, as long as the data had not yet been anonymized. Parents were informed of their ability to withdraw their child or their child’s data from the study at any time. They were assured that identifying data would only be accessible to the researchers and that this information would be deleted no later than five years after the study. Lastly, the parents were provided with a link to the data protection regulations followed during the study.

### Item analyses

Item analyses will be conducted using Rasch measurement models, as the KIDSCREEN questionnaires were originally developed and validated using Rasch measurement models, and thus the Danish language version is expected to meet the same measurement requirements.

#### The Rasch model

The Rasch model (RM) [55] is the first and statistically most parsimonious in what has become a family of item response theory models [56]. The RM was first developed for dichotomous items and later generalized to ordinal items and parameterized in different ways [57–60]. In the present study, we use the partial credit parametrization of the RM for polytomous items [58]. As the requirements for the dichotomous and the ordinal Rasch models are the same [61], we use RM for Rasch model in the remainder of the article. The RM is often considered to be the gold standard within scale validation, because scales fitting a RM not only are criterion-related construct valid according to Rosenbaum [62] definition, but also results in a number of desirable properties of the scale in question [63, 64]:

1. *Unidimensionality*: the scale measures a single construct, here well-being.
2. *local independence (no local dependence*, *no LD)*: items are conditionally independent given the latent variable, meaning that the response to a well-being item depends only on the level of well-being and not on responses to any of the other well-being items.
3. *absence of differential item functioning (no DIF)*: Items and exogenous variables are conditionally independent given the latent variable, meaning that the response to any well-being item depends only on the level of well-being and not on the child’s membership of subgroups such as for example age, gender, class level of the children, etc. Establishing absence of DIF is of great importance when using latent variable measurement scales in social and educational studies. If present, DIF would mean that the obtained scale values on the KIDSCREEN-10 were to some degree dependent on background variable(s) and not only on the actual level of the child’s/adolescent’s well-being. Accordingly, the KIDSCREEN scores would be artificially low or high for some groups and thus not directly comparable for these groups. In addition, if the effect of the DIF is sufficiently large, this might also carry implications for screening purposes, as the DIF might also cause individuals to have inflated or deflated scores to a degree, which would place them on the wrong side of a screening cut point.
4. *sufficiency of both the sum score and the estimated Rasch score*: information on membership of subgroups or response patterns is not needed to assess the level of well-being using the simple summed score. Establishing sufficiency of the sum score is considered important when using the KIDSCREEN, as this allows for easy use by practitioners choosing not to use the person parameter estimates (sometimes denoted Rasch scores), as well as development of norms based on both sum scores and person parameter estimates.
5. *Specific objectivity of measurement by the scale*: The comparison of items is not biased by the selection of persons and that the comparison of persons is not biased by the selection of items.

The properties of *sufficiency* and *specific objectivity* are properties only of scales fitting Rasch models.

#### Graphical loglinear Rasch models

Previous construct validity studies of the KIDSCREEN-10 in other language versions have shown evidence of DIF and LD. Therefore, we expect to also use graphical loglinear Rasch models (GLLRM), as in the study by Müller and Hoti [28], to account for such departures from the pure Rasch model. GLLRMs are extended and generalized Rasch models proposed by Kreiner and Christensen [65–67] to avoid eliminating items from relatively small sets of items. In GLLRMs, items may be locally dependent if the strength of the association between dependent items is constant across all levels of the latent variable. Likewise, items may function differently in different subpopulations defined by background variables if the direct effect of the background variable on the item is constant across all levels of the latent variable. Kreiner and Christensen referred to DIF and LD satisfying these requirement as uniform DIF and uniform LD and claimed that Rasch models with uniform DIF and uniform LD provide *essentially* valid and objective measurement [67, 68]. The adding of uniform LD and/or DIF to Rasch models was originally proposed by Kelderman [69] in loglinear Rasch models. GLLRMs differ from Kelderman’s log-linear Rasch model as they insert the log-linear Rasch model in multivariate chain graph models, which include background variables.

In chain graph models, nodes represent variables and connections/edges between nodes illustrate associations among variables. Missing edges or arrows between nodes means that the variables are *conditionally* independent, given the remaining variables in the model. An arrow connecting two variables in a chain graph model may refer to a causal relationship and undirected edges means that the variables are conditionally dependent without causality assumed (see [70] for further details on graphical models). Items and other variables in GLLRMs follow the same rules. Items that are not connected by an edge in a GLLRM graph are conditionally independent given the latent variable (i.e. items are locally independent). Likewise, if there is no arrow or edge between an item and a background variable it means that they are conditionally independent given the latent variable and the other variables in the model. In other words, this implies absence of DIF. Items are connected to the latent variable to indicate that the relationship is causal.

#### Sufficiency and specific objectivity of measurement

The properties of sufficiency of the scores and specific objectivity belong exclusively to the RM [71], but measurement by GLLRMs may be regarded as essentially objective and valid. Also, if the GLLRM only includes LD the sufficiency of the scores is retained. Departures from the RM in the form of uniform LD may, however, lead to reduced reliability, as the usual estimation of Cronbach’s alpha tends to overestimate the lower bound of reliability when items are not locally independent [71]. If the GLLRM includes DIF, the score is no longer a sufficient statistic for the person parameter estimates across subgroups, but only within subgroups, as further information is needed to deal with the DIF in subsequent analysis using the score [67]. Departures in the form of uniform DIF will thus affect the validity of the scale in question and limit its use in further studies or analyses involving comparisons if scores are not equated across groups showing DIF [68, 72]. In such cases, we will provide DIF-equation tables for the sum score. We will also provide conversion tables to enable user of the scale to use sum scores or person parameter estimates in subsequent analyses or assessments.

#### Strategy of analyses and statistical tests

A rigorous test of the fit of a RM or a GLLRM to a set of items making up a single scale includes the following steps:

- Overall test of homogeneity of item parameters across low and high scoring groups.
- Overall tests of invariance relative to important background variables.
- Tests of no DIF for all items relative to the same background variables.
- Tests of local independence for all item pairs (no LD).
- Fit of the individual items to the RM.

The steps will not necessarily be taken in the order presented above. If evidence of LD or DIF turns up, log-linear interactions will be added to the model and the steps repeated until no further evidence is disclosed.

When the final step has been taken the following steps will conclude the analysis:

- Assessment of standard errors and bias of measurement.
- Evaluation of targeting and reliability relative to the current study population.

The fit of individual items to the RM or a GLLRM will be tested by comparing the item-rest-score correlations with the expected item-restscore correlations under the model [66, 73]—restscores are total scores where the item in question has been excluded [74]. The overall homogeneity across score groups, as well as overall invariance, will be tested by Andersen’s [75] conditional likelihood ratio tests (CLR) comparing item parameters in subpopulations defined by score groups or exogenous variables for DIF analysis. Local independence of items and DIF will be tested using Kelderman’s [69] likelihood-ratio test, and if evidence against these assumptions is discovered the magnitude of the local dependence of items and/or DIF will be informed by partial Goodman-Kruskal gamma coefficients [76] conditional on the restscores [66]. Analyses of overall invariance and DIF will be done in relation to four exogenous variables: Language spoken in the home (Danish, other than Danish), School (public school, private school), Gender (boy, girl), and Grade (grade 5, grade 6, grade 7, grade 8) (c.f. Table 1).

All the test statistics tests whether the item responses comply with the expectations of the model, and all results will be evaluated in the same way; significant p-values signifies evidence against the model. Following the recommendations by Cox and colleagues [77], we will evaluate p-values as a continuous measure of evidence against the null hypothesis of no difference, while distinguishing between weak (p < 0.05), moderate (p < 0.01), and strong (p < 0.001) evidence against the model, rather than applying a deterministic critical limit of 5% for p-values. Furthermore, we will use the Benjamini-Hochberg [78] procedure to control the false discovery rate (FDR) due to multiple testing to reduce the amount of false evidence against the model.

In case of fit to the pure Rasch model, reliability will be estimated as Cronbach’s alpha, and otherwise Hamon and Mesbah’s [71] adjusted Monte Carlo-based estimation, which takes into account any local dependence in a GLLRM, will be used. Targeting of the latent scale will be assessed numerically with two indices [73]: the test information target index (the mean test information divided by the maximum test information) and the root mean squared error target index (the minimum standard error of measurement divided by the mean standard error of measurement). Both indices should have a value close to one. We will also estimate the target of the observed score and the standard error of measurement of the observed score.

### Development of norms

Norms will be developed after completing of the item analyses so that we might develop norm tables based on both raw scores and the person parameter estimates from the Rasch analyses (i.e. Rasch scores), as done for the original and other newly developed international norms for the KIDSCREEN-10, e.g. [49] with new German norms, However, we will be able to go one step further and adjust sum scores for any uniform DIF evidenced in the analyses by graphical loglinear Rasch model, and thus we will also be able to generate norms based on the sum scores which are free of DIF effects. We will provide percentiles, T and Z scores based on (DIF-equated) sum scores and the Rasch scores. In addition, we will develop norms with a higher degree of differentiation compared to the international norms, as we divide norms into single grade levels rather than grade or age intervals, as done previously. This will facilitate a culturally appropriate and more differentiated assessment than what is available in the various European norms, where norms are provided for gender and age intervals of 3–4 years [27].

### Software

All item analyses were conducted using the DIGRAM software package, as the implementation of the RM, as well as the graphical loglinear Rasch model in this package, provides formal tests for global fit, unidimensionality, DIF and LD, while adjusting for false discovery rate due to multiple testing [79–81]. Norms will be developed using standard statistical software (i.e. SPSS).

## Results

### Initial analyses

Initially analyses were attempted using the complete sample of students in a single analysis, and using four subsamples of students defined by grade levels. However, these analyses all provide massive, but inconclusive, evidence against fit to the Rasch model (i.e., evidence against item fit, local independence of items, overall homogeneity and evidence of DIF), which could not be resolved by attempts to model GLLRMs and test fit of the response data to these. S2 File contains raw output documenting first the initial evidence against fit to the Rasch model, followed by evidence against fit to the last GLLRM including uniform DIF and local dependence interaction terms analysed (i.e., after this step further analyses were not possible as model became too complex). The poor initial results could have been taken to be the “end of the road” for the Danish KIDSCREEN-10, concluding that it was not a valid unidimensional scale fitting the Rasch model, as claimed for other language versions of the KIDSCREEN-10 (cf. the above). Large-sample validation studies had not been conducted since the initial development of the KIDSCREEN-10, and in the development study data had been collected with the KIDSCREEN-52 and then reduced to the 10 KIDSCREEN-10 items. The KIDSCREEN-10, thus had not previously been validated in its own right to show its validity for the purpose of population and other large-scale studies. Previous studies have succeeded in conducting item analyses using Rasch models and even graphical loglinear Rasch models, though with smaller samples than ours. Thus, we suspected that the issue might be that the KIDSCREEN-10 data could fit Rasch models with smaller samples when taking into account DIF and/or local response dependence, but not for larger samples. We thus amended our strategy of analyses to be able to detect at which sample size the analyses of the KIDSCREEN-10 failed in order to attempt to uncover more precisely the measurement issues causing this failure and the severity of them.

### Amended strategy of analyses

Firstly, we defined a uniform random variable (Z) with 40 categories in order to achieve approximately 2,5% of cases in each category (S1 Table). Subsequently, Z was categorised into seven categories with an increasing number of cases in each category, aiming for approximately 2,5%, 5%, 7,5%, 10%, 12,5%, 20% and 40% (Table 2). Lastly, the dataset was split into seven partial datasets defined by Z, and each dataset was analysed separately starting with the smallest one. We followed the general strategy of analyses for the first and smallest subsample, and for each of the following sample, we first tested whether there was fit to the model for the previous and smaller sample. If this was not the case, we followed again the general strategy of analyses until we achieved fit to a GLLRM.

**Table 2:** The distribution of cases in the seven new samples to be analysed.

Sample	Includes Z-categories	n	percentage of total sample
1	1	214	2.6
2	2–3	413	5.1
3	4–6	630	7.7
4	7–10	835	10.2
5	11–15	1050	12.9
6	16–23	1651	20.2
7	24–40	3378	41.3

### Item analyses by Rasch and graphical loglinear Rasch models

In the following, we show the overall results of the analyses of each of the random samples in the form of the resulting graphical loglinear Rasch models, as these illustrate issues with both locally dependent items and DIF. All additional output relating to item fit, overall invariance, global homogeneity, any evidence of additional local dependence among items and/or DIF, as well as evidence for the DIF and local dependence included in the GLLRM, are provided in S3 File, as they are too extensive to place within the article itself.

In order to relay the many results as simply as possible, we use letters as short variable names for both items and exogenous variables in the graphical loglinear Rasch models. Items are thus named Kid1 to Kid10. Item Kid4 is reverse scored in accordance with the scoring manual. The exogenous variables are named Lang (language spoken in the home), School (type of school), Sex (gender), and Grade (grade level).

#### Model 1

Analyses of the smallest random sample (n = 214) showed that initially item Kid3 (*have you been sad*?*)* was eliminated as the item-restscore correlation was very low (p < .0001) (i.e. no item fit) even after including interaction terms for local dependence between items Kid3 and Kid4 (*have you felt lonely*?*)* and DIF for item Kid3 with gender. We, thus decided to eliminate item Kid3, as this item appears to reflect what could be a consequence of any of the remaining nine items rather than just an indicator of HRQoL. Müller and Hoti [28], using the same test of item fit, as we use in this study, also reported a lack of fit of item Kid3 as well as item Kid1 (*having felt fit and well*) for the German language version of the KIDSCREEN-10, but chose to keep the two items in the scale. Gong et al. (2021) on the other hand eliminated both item Kid3, Kid4 and Kid5 due to non-fit.

**Fig 2:**
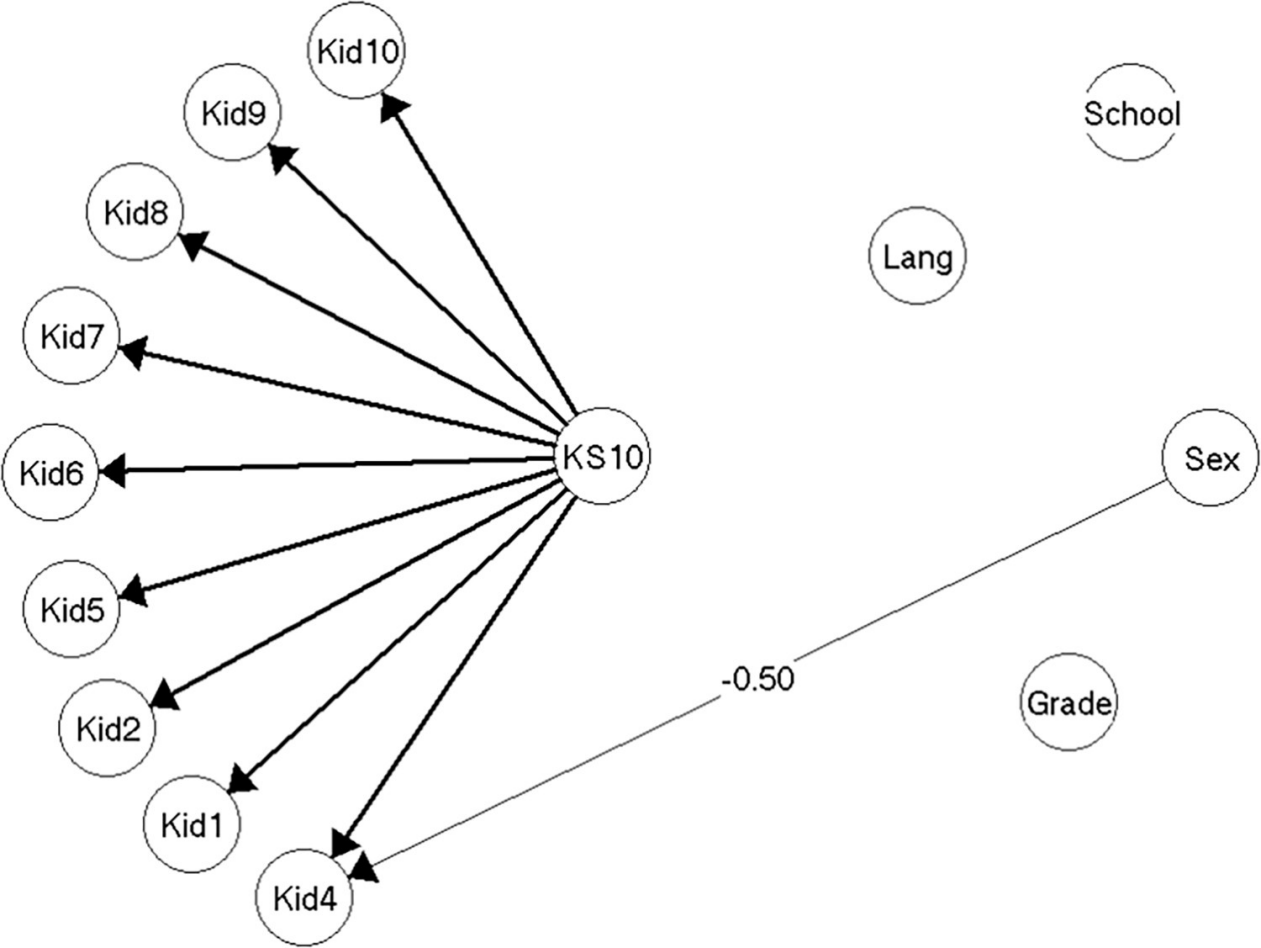
Resulting model 1 (n = 214).

The model for the nine remaining items (item Kid3 eliminated) did not reveal any issues with local dependence of items, but revealed strong gender DIF for item Kid4 (see above), so that girls systematically scored this item higher than did boys given the same level of HRQoL (*note that Kid4 is reverse scored*). The model 1 (Fig 2) was accepted without problems (see S3 File, section on model 1).

Even though model 1 only included gender DIF on a single item, the effect of the DIF in model 1 was large enough to result in adjusted individual scores for girls with more than 0.5 points in some cases. If scores were not equated for DIF, it would result in a test bias of 0.40 points for girls on the mean score (S3 File, section on model 1).

#### Model 2

**Fig 3:**
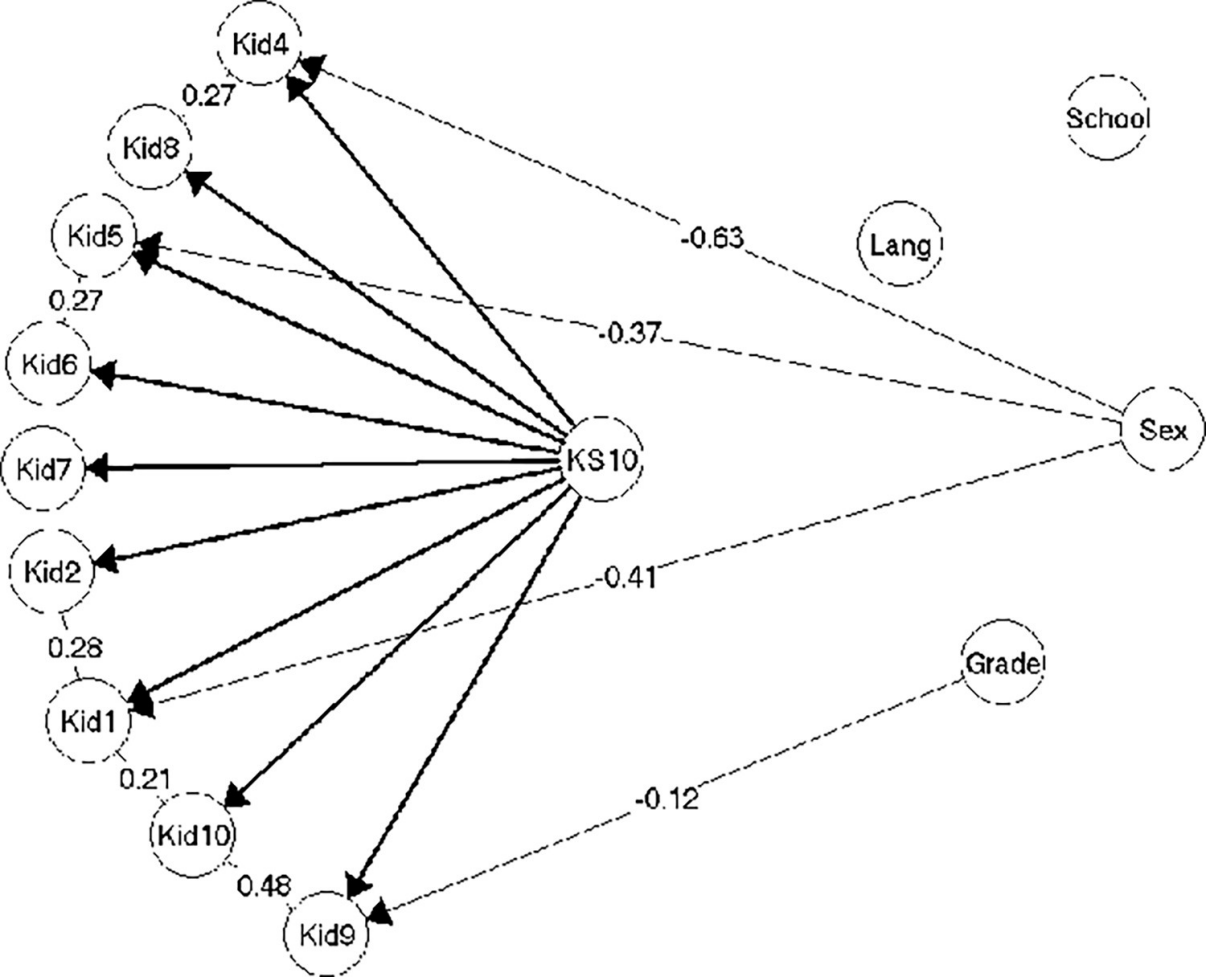
Resulting model 2 (n = 413).

Already with the second smallest sample (n = 413), substantial amounts of evidence of local dependence appeared (Fig 3). Three pairs of locally dependent items reflected the dimensionality in KIDSCREEN-27; Kid1-Kid2, Kid5-Kid6, Kid9-Kid10, while four of the locally dependent item pairs were identical to findings reported in Müller and Hoti [28]; Kid1-Kid2, Kid5-Kid6, Kid4-Kid8, Kid9-Kid10. The fifth and last pair of locally dependent items was Kid1-Kid10, suggesting that the association between a general sense of well-being and the ability to be attentive goes beyond what can be explained by HRQoL.

In addition to local dependence among items, the evidence of DIF relative to gender was expanded to include three items; Kid1, Kid4 and Kid5. As in model 1, girls were more inclined to endorse *having felt lonely* (Kid4), while it is the boys who are more inclined to endorse *having felt fit and well* (Kid1) and *having had enough time for themselves* (Kid5) given same levels of HRQoL. In addition, there was weak evidence of DIF for item Kid9 (*having been able to pay attention)* relative to grade level, so that pupils were more inclined to endorse this item the lower the grade they were attending given the same level of HRQoL. The model 2 (Fig 3) was accepted without problems (see S3 File, section on model 2).

The gender and grade level DIF in model 2 had a very large effect in terms of test bias. Thus, if scores were not equated for the DIF, the test bias on the mean scores of girls in the different grades would be between -1.58 to -1.75, while much less for boys, resulting in the adjustments of the individual scores with as much as 2,35 score points (girls in grade 6) (S3 File, section on model 2).

#### Model 3

In the analyses of the third random sample (n = 630), almost the same item pairs were found to be locally dependent as in model 2. The discrepancies were: The local dependence between items Kid1 and Kid10 was replaced by a weak local dependence between items Kid2 and Kid10. An equally weak local dependence between items Kid6 and Kid10 was also introduced. Both of these local dependencies were so weak that they have no practical implications (γ = 0.02 in both cases).

The gender DIF for items Kid1 and Kid5 in model 2 disappeared again in model 3, and only the gender DIF for item Kid4 (*having felt lonely*) and the grade level DIF for item Kid9 (*have you got on well at school*?) remained.

**Fig 4:**
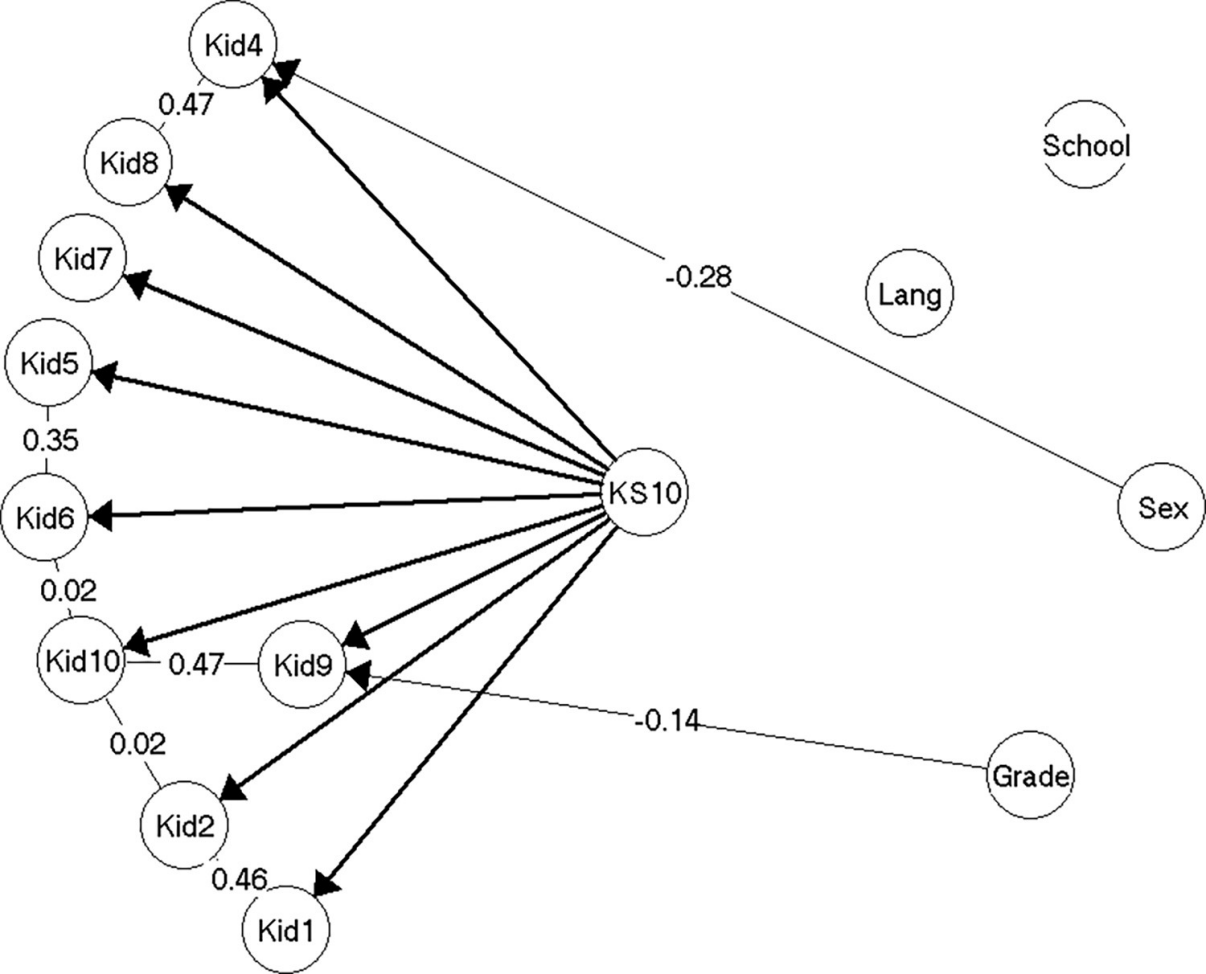
Resulting model 3 (n = 630).

Model 3 (Fig 4) was not entirely accepted, as there was very weak evidence against overall invariance across all items with regard to gender and grade level, thus suggesting there might be more DIF related to these variables than what we were able to discover (see S3 File, section on model 3).

The gender and grade level DIF in model 3 was less extensive and thus also has a smaller effect in terms of test bias, than the DIF in model 2. Thus, if scores were not equated for the DIF, the test bias on the mean scores would at most be -0.28 (girls in the 5th and 8th grade), due to the equally smaller adjustments of the individual scores (maximum 0.58 score points, girls in grade 8) (S3 File, section on model 3).

#### Model 4

With the analyses of the fourth sample (n = 825), the weak local dependency interaction between items Kid6 and Kid10 disappeared again, while the weak local dependence between items Kid2 and Kid10 became stronger, compared to model 3.

With regards to DIF, the gender DIF for item Kid4 (*have you felt lonely*?) present in model 3 remained, while the gender DIF for item Kid5 discovered in model 2 turned up again. In addition, evidence of new gender DIF was found for item Kid9, so that girls are more inclined to endorse *having got on well at school* compared to boys, given the same levels of HRQoL. Lastly, there was strong evidence of weak DIF relative to the language spoken in the home for items Kid4 and Kid6. For both items the DIF meant that children where the language spoken in the home is Danish were slightly more inclined to say that they *had been able to do the things that they wanted in their free time* (Kid6) and *that they had felt lonely* (Kid4) compared to children where the language spoken in the home was not Danish, given the same levels of HRQoL.

**Fig 5:**
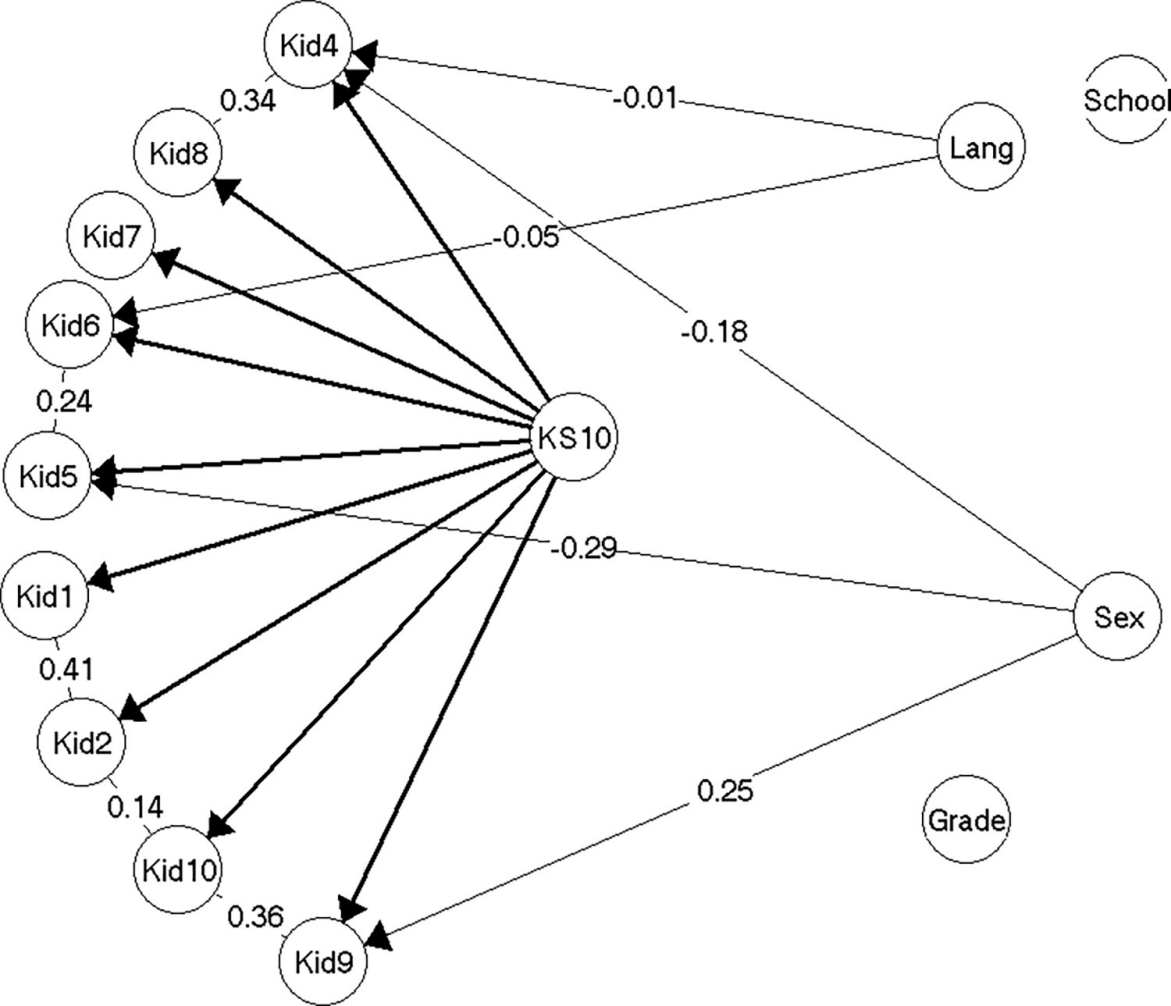
Resulting model 4 (n = 825).

As with model 3, model 4 (Fig 5) was not entirely accepted. For model 4, we found weak evidence against overall invariance across all items with regard to language in the home (K). This suggested that there might be more DIF related to the language spoken in the home compared to what we discovered, or that this result was a type I error. In addition, there was very weak evidence against the fit of Kid1 (*having felt fit and well*) in model 4, as well as very weak evidence against overall homogeneity and overall invariance across all items with regard to grade level (see S3 File, section on model 4).

The gender and language DIF in model 4, did not have a large effect in terms of test bias, even though adjustments of scores at the individual level were quite high in some cases. Thus, if scores were not equated for the DIF, the test bias on the mean scores would at the most be -0.34 (girls with another language than Danish being spoken in the home), even though individual adjustments were as high as 1.36 score points (again with another language than Danish being spoken in the home) (S3 File, section on model 4).

#### Model 5

**Fig 6:**
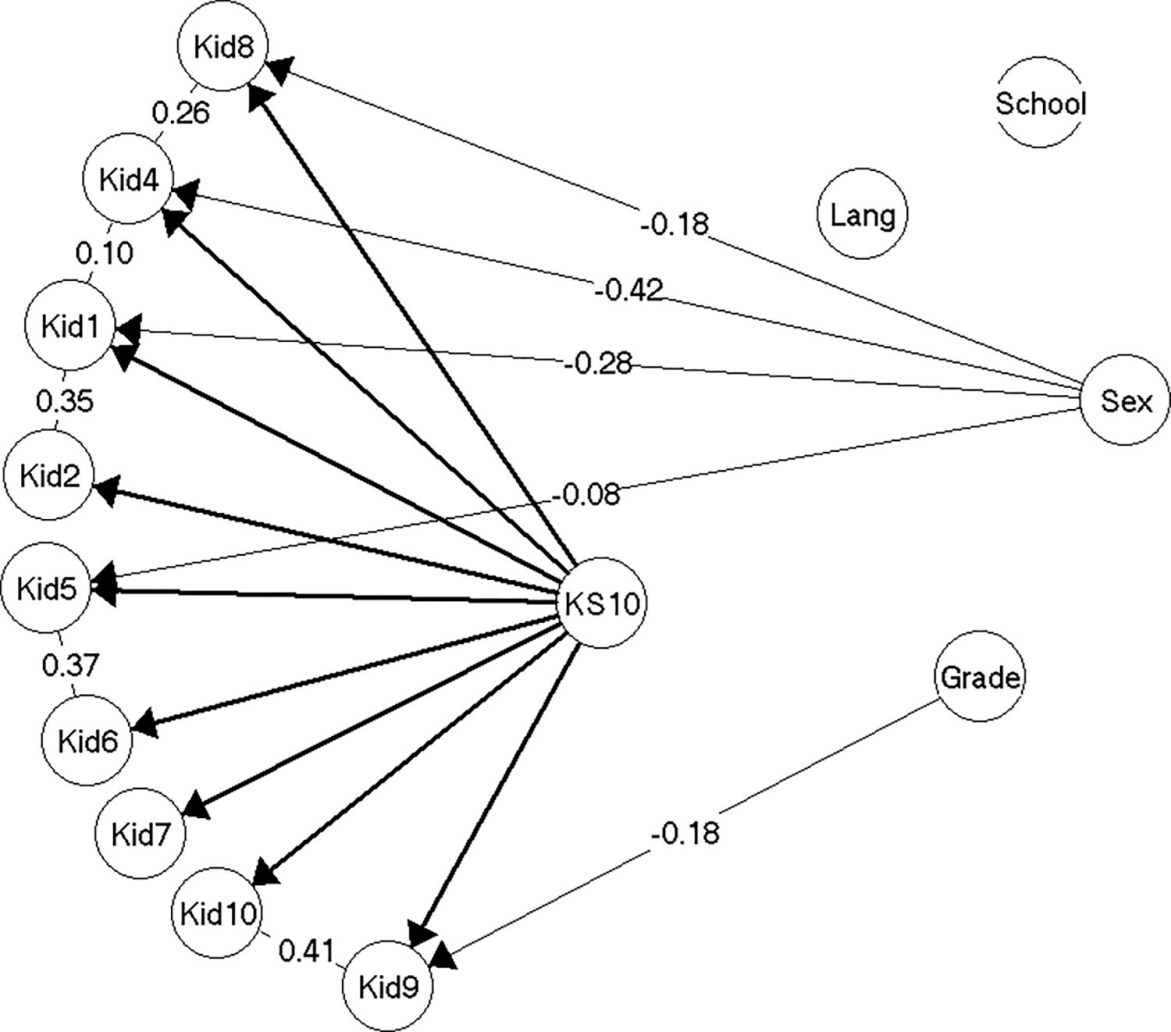
Resulting model 5 (n = 1050).

The analyses of the fifth sample (n = 1050) resulted in the disappearance of the weak local dependency interaction between items Kid2 and Kid10 disappeared again, and instead there was evidence of weak local dependence between items Kid1 and Kid4. The remaining local dependence present in model 4, remained in model 5 (Fig 6).

The evidence of gender DIF for items Kid4 and Kid5, from model 4, prevailed in model 5, while the evidence for gender DIF for item Kid9 and the very weak language DIF for item Kid4 and Kid6 disappeared (Fig 6). In addition, evidence for the gender DIF for item Kid1 and the grade level DIF for item Kid9, which were present in model 2 and 3, reappeared. Lastly, new evidence of additional gender DIF for item Kid8, so that so that girls are more inclined to endorse *having had fun with their friends* compared to boys, given the same levels of HRQoL, was introduced in model 5.

For model 5, the only evidence against fit to the GLLRM in Fig 6, was the very weak evidence against the fit of item Kid1 (*having felt fit and well*), which was also seen in model 4 (see S3 File, section on model 5).

The size of the fifth sample was close to the sample size in the study by Müller and Hoti [28], and the evidenced local dependence among items was almost identical across the two studies. However, our model 5 was more complex in terms of more gender DIF, as this was present for four items versus just for a single item in the GLLRM in Müller and Hoti [28]. However, in Müller and Hoti’s study, the tests for overall invariance across gender and citizenship for the set of items rejected invariance, thus indicating the presence of more DIF than was discovered and as such evidence against the model (Table 4 in [28]).

The gender and grade level DIF in model 5 had a large effect in terms of test bias, as in model 2. Thus, if scores were not equated for the DIF, the test bias on the mean scores of girls in the different grades would be between -1.12 to -1.62, while much less for boys, resulting for the adjustments of the individual scores with as much as 1.96 score points (girls in grade 7) (S3 File, section on model 5).

#### Model 6

**Fig 7:**
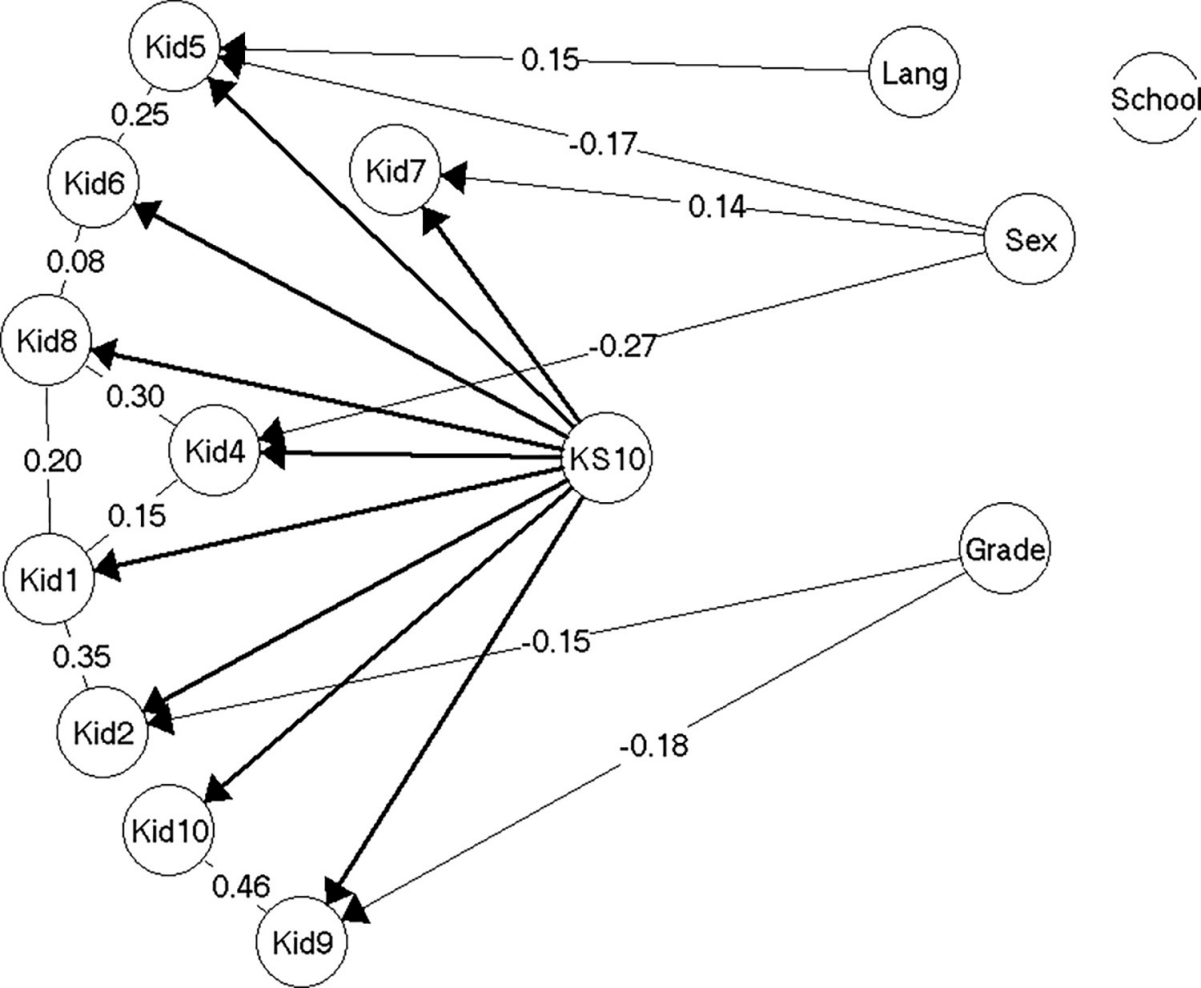
Non-fitting model 6 (n = 1651).

With the analyses of the sixth sample (n = 1651), we were not able to establish fit of the item response set to a GLLRM. Working forward from model 5, we reached a complex model, with evidence of extensive local dependence among items as well as DIF for a total of five of the nine items (Fig 7), for which fit could not be established, however. There was only weak evidence against fit of items Kid1, Kid7 and Kid9 to the model. However, there was strong evidence against overall homogeneity, as well as strong evidence against overall invariance of the full set of items across language groups, school groups and grade levels, suggesting that we had not discovered all DIF in the model (S3 File, section on model 6). In addition, the model did not converge, which made it impossible to proceed with towards a more complex model, and we had to abandon the analyses of the sixth sample at this point.

The sample size for model 6 was close to Gong et al.’s sample size of 1830 [33]. They were able to conduct their analyses and reach a model, but only so after eliminating three items (including items with DIF) and collapsing response categories.

### Norms

The planned generation of Danish norms was not realizable, as it had not been possible to achieve fit to the Rasch or graphical loglinear Rasch models and thus not possible to generate the valid sum scores nor valid estimates of the person parameters needed.

## Discussion

The purpose of this study was to, examine the criterion-related construct validity of the Danish language version of the KIDSCREEN-10 questionnaire as well as its psychometric properties using Rasch models and to construct Danish norms based on the resulting person parameter estimates from the Rasch models.

KIDSCREEN-10 is broadly used, internationally as well as in Denmark, despite the fact that no studies have previously shown compliance with the properties of the Rasch model, as claimed by the group of developers. In Denmark, no studies have previously attempted to validate the Danish language version, neither for use in large scale population studies, as is the intended use, nor for smaller n intervention studies with vulnerable children and adolescents. Despite these facts, the Danish KIDSCREEN-10 is recommended for the latter use by the National Board of Social Services in Denmark.

In the present study, we chose to eliminate item Kid3 (*have you felt sad*?) early in the extensive regime of analyses. This might be a point of criticism. However, Gong et al. [33] also eliminated this item, and the remaining studies, including the development study, have all reported varying degrees of measurement issues with item Kid3 [27, 28, 33, 34, 43]. The KIDSCREEN-10 (and the other KIDSCREEN questionnaires) were developed to comply with Rasch model properties, and is being used as such with norm data based on the assumption that they fit Rasch models. Fit to the pure Rasch model has not been replicated in independent studies during the almost twenty years since the release of the KIDSCREEN questionnaires. Three studies conducted with three different language versions of the KIDSCREEN-10 have found problems with fit to the Rasch model in various ways. Erhart et al. [43] investigated 15 language versions, and presumably only assessed fit of the individual items to the Rasch model. While items were found to fit in most countries, this was not the case for item Kid9 (*have you got on well at school*?) in Greenland, Russia or Macedonia, and also not the case for item Kid3 (*have you felt sad*?) for Russia. Velez et al. [34] reported no issues with item fit with the Spanish version used in Columbia, but they found evidence of DIF relative to socioeconomic status for item Kid3 (*have you felt sad*?) and item Kid4 (*have you felt lonely*?). Gong et al. [33] reported misfit for items Kid3, Kid4, and Kid5 (*have you had enough time for yourself*?), as well as age DIF for items Kid3 and Kid4, for a Chinese version.

In the studies by Erhart et al., Velez et al., and Gong et al. [33, 34, 43] item fit was assessed using unconditional infits and cut-off values of 0.7 and 1.3. In comparison, the development study [27] used the narrower interval of 0.8 to 1.2 to assess the fit of individual items, and thus the development study used stricter criteria than the later independent studies. Despite this the later studies have not been able to replicate the development study. Furthermore, unconditional infits have been found to provide erroneous results with sample sizes over 250 and that the rule-of-thump of infits between 0.7 and 1.3 is not reliable with unconditional infits [82], and thus the previously mentioned results might not be entirely correct in their results concerning items fit.

Müller and Hoti [28] and this study are, to our knowledge, the only studies having used unconditional infits to assess item fit for which the rule-of-thump of 0.7 to 1.3 for item fit has been found to be useful. However, both Müller and Hoti [28] and we have used Monte Carlo bootstrapping to calculate valid p-values, as recommended by [82], and we have also used other fit statistics for item fit. Thus, the most reliable result concerning item fit should be found in these two studies. Furthermore, Müller and Hoti [28] and the present study, are the only studies who have included analysis of conditional independence of items, and found that this is not fulfilled, and the only studies applying graphical loglinear Rasch models in an attempt to achieve fit to a model with the full set of 10 items for the KIDSCREEN-10 child and adolescent version.

In the present study, it was not possible to fit the data to a Rasch or a graphical loglinear Rasch model, once we attempted to analyse just over 600 cases. Evidence of additional local dependence, differential item functioning and lack of fit of individual items persisted. Furthermore, there was a lack of overall invariance and homogeneity, which could not be resolved. At just over 1600 cases attempts to resolve a model failed all together.

The measurement issues discovered with the various smaller sample sizes were not trivial. There was evidence of lack of fit for item Kid3 (*have you felt sad*?*)*. Even, when this was eliminated, there was also evidence of substantial local dependence, as well as gender and grade level DIF leading to substantial test bias, if not dealt with. This means that users of the Danish language version with smaller samples, would have to eliminate item Kid3 and subsequently equate scores for DIF, in order to obtain scores that are comparable across subgroups. This can be done using the DIF-equating Tables in the S3 File, but as the models differ somewhat for the different samples with regard to DIF, it is not an easy choice to determine whether to use results from the smallest random sample or the second smallest sample. However, as the models differed for the two smallest samplesit is not trivial to account for local dependence and DIF.

As it was not possible to reach an acceptable model for a sample size appropriate for producing norms, no Danish norms could be produced. This means that users’ only choice is to use culturally inappropriate and time-wise obsolete norms, in the form of the old common European norms from the development phase [27], or the newer German norm [49]. Furthermore, in using the foreign norms, users would be forced to include the eliminated question of having felt sad (item kid3), as this is included in the norm scores.

Future studies will need to establish the extent to which the negative results in the current study extend to the parent-proxy version of the KIDSCREEN-10 or if it can be used validly in the Danish context. As proxy versions are typically used for smaller intervention studies, we recommend that user of the Danish parent-proxy version carefully investigate whether this complies with the Rasch model requirements, as intended by the developers [27]. The Danish KIDSCREEN-27 consists of five subscales, from which the items from the KIDSCREEN-10 were drawn. While we did not find evidence of multidimensionality as such in the current study, the pattern of locally dependent items partially reflected the subscale structure of the KIDSCREEN-27. Thus, it is more likely that each subscale of the KIDSCREEN-27 complies with Rasch model or loglinear Rasch model requirements, most likely the latter, as DIF is to be expected. However, as the five KIDCSREEN-27 subscales are comprised of items from the ten subscales of the KIDSCREEN-52, they might lack unidimensionality and some items will more than likely have other measurement issues such as DIF. Users of both the Danish KIDSCREEN-27 and KIDSCREEN-52 should therefore also be advised to ensure that measurement by each subscale, included in these, comply with Rasch (or at least loglinear Rasch) model requirements.

## Conclusions

In conclusion, we cannot recommend the use of the Danish language version of the child/adolescent self-report KIDSCREEN-10 questionnaire for population-level studies, even though this is the intended use for the KIDSCREEN questionnaires, as we were not able to establish fit of the data to the Rasch model for any substantial sample size. Though fit to a Rasch model was achieved for small sample sizes, the ambiguous nature of the DIF-results, also means that we cannot recommend use of The Danish KIDSCREEN-10 child/adolescent version for small-scale intervention or other similar studies with vulnerable (or non-vulnerable) children/adolescents either.

In addition, as it was not possible to reach an acceptable model for a sample size appropriate for producing norms, no Danish norms could be produced for the KIDSCREEN-10.

## Supporting information

S1 Table The uniform random variable Z (sample) distribution across the 40 categories. (PDF)

S1 File Detailed description of the sampling design and sampling results of the project data was drawn from. (PDF)

S2 File Documentation of the initial evidence against fit the Rasch model, and evidence against fit for the last analysis attempted on the full sample. (PDF)

S3 File Additional results for each of the models resulting from analyses of the random samples. (PDF)
