# Supplementary material for: Measuring child and adolescent well-being in Denmark: Validation and norming of the Danish KIDSCREEN-10 child/adolescent version in a national representative sample of school pupils in grades five through eight
Source: PLoS One. 2023 Sep 8;18(9):e0291420. doi: 10.1371/journal.pone.0291420 (PMC10490965; doi:10.1371/journal.pone.0291420)
Supplement: S1 Table — (PDF) [file pone.0291420.s001.pdf]

S1 Table. The uniform random variable Z (sample) distribution across the 40 categories.

Z	Count	Pct	CumPct
1	214	2.62	2.62
2	199	2.44	5.05
3	214	2.62	7.67
4	209	2.56	10.23
5	205	2.51	12.74
6	216	2.64	15.38
7	185	2.26	17.65
8	207	2.53	20.18
9	229	2.80	22.98
10	214	2.62	25.60
11	211	2.58	28.19
12	216	2.64	30.83
13	218	2.67	33.50
14	206	2.52	36.02
15	199	2.44	38.45
16	213	2.61	41.06
17	188	2.30	43.36
18	216	2.64	46.00
19	227	2.78	48.78
20	214	2.62	51.40
21	202	2.47	53.87
22	188	2.30	56.17
23	203	2.48	58.66
24	188	2.30	60.96
25	218	2.67	63.63
26	179	2.19	65.82
27	192	2.35	68.17
28	193	2.36	70.53
29	186	2.28	72.81
30	193	2.36	75.17
31	198	2.42	77.59
32	214	2.62	80.21
33	201	2.46	82.67
34	207	2.53	85.20
35	186	2.28	87.48
36	189	2.31	89.79
37	210	2.57	92.36
38	203	2.48	94.85
39	211	2.58	97.43
40	210	2.57	100.00
