# Supplementary material for: Measuring child and adolescent well-being in Denmark: Validation and norming of the Danish KIDSCREEN-10 child/adolescent version in a national representative sample of school pupils in grades five through eight
Source: PLoS One. 2023 Sep 8;18(9):e0291420. doi: 10.1371/journal.pone.0291420 (PMC10490965; doi:10.1371/journal.pone.0291420)
Supplement: S1 File — (PDF) [file pone.0291420.s002.pdf]

S1 File. Detailed description of the sampling design and sampling results of the project data was drawn from

Introduction

The data for this article originates from the project *Children and young people's reading 2021* [Børn og unges læsning 2021], which examines children's reading habits and experiences at four grade levels in Danish schools and so it has four target populations – students enrolled in the fifth, sixth, seventh, and eighth grade (Hansen et al., 2022). The overall purpose was to provide generalizable insights into the reading habits of each of the four target populations. Importantly, the study's questionnaire also included the KIDSCREEN-10 items, which makes the data relevant for this article.

Children and young people's reading 2021 utilized a two-stage random sample design, with a sample of schools drawn as a first stage and one intact class of students selected from each target grade in participating schools as a second stage. Intact classes of students were sampled rather than individuals to secure less disruption to the school's day-to-day business. For practical reasons, the selection of schools was split into two separate sampling plans. In one sampling plan, schools were selected to participate with a class in the fifth and sixth grade from a list of schools with students enrolled in the two target grades. In the other sampling plan, schools were sampled to participate with a class in the seventh and the eighth grade from a list of schools that had students enrolled in both the seventh and eighth grade.

The data was collected in Autumn 2021 – from the 15th of September to the 17th of November – using online surveys that children answered in a classroom setting. The data collection resulted in completed responses from 2119 children in the fifth grade (from 115 schools), 2118 children in the sixth grade (114 schools), 1976 children in the seventh grade (105 schools), and 1958 children in the eighth grade (106 schools).

Target population

Children and young people's reading 2021 had four target populations, i.e. children enrolled in the fifth, sixth, seventh, and eighth grade. In the Danish school system, compulsory education consists of ten years of primary and lower secondary education, including one pre-school year (ISCED level 0) and years 1 – 9 (grades). Children normally start in pre-school the year they turn six, and they enroll in the first grade the following year (i.e. first year of ISCED Level 1). Consequently, when children are in the fifth grade, they are in their fifth year of schooling and most of them are in the age range of 10 to 11. S1-1 Table provides details about the four target populations.

S1-1 Table. Details about target populations

Target population	Years of schooling	Approx. age range	Population size
5th grade	5	10-11	68806
6th grade	6	11-12	67880
7th grade	7	12-13	69329
8th grade	8	14-15	68222

Notes. Information about the population size is based on data about all Danish schools from the school year 2018/2019. It was received from the Danish Departmental Agency for IT and learning [STIL; Styrelsen for IT og Læring] (Pettersson et al., 2022).

All students enrolled in a target grade were part of their respective target population, regardless of their age. As students were sampled in two stages, first by random selection of schools and then random selection of a class from participating schools, it was also necessary to identify and define eligible schools. Essentially, all schools were eligible for the study, if they had students enrolled in the respective target grade, regardless of the type of school.

Sampling frame
The project used two sampling frames. One with all schools that had students enrolled in the fifth and the sixth grade (N=1599), and another with schools that had students enrolled in the seventh and the eighth grade (N=1319).

The two sampling frames were constructed based on data from the Danish Departmental Agency for IT and learning [STIL; Styrelsen for IT og Læring] in Spring 2020 (Pettersson et al., 2022). STIL is an agency supporting the Department of Education in Denmark and it keeps an updated database of all schools in Denmark.

Each sampling frame was structured as a spreadsheet containing a single row per school. Every row contained important information such as a unique identification number, contact information and the values of the stratification variables for the school (i.e. school type) and the school measure of size. For a full overview, both sampling frames included the following information:

- School ID
- School type: Public or private
- School size: Number of students in the school
- Grade size: Number of students in each target grade
- School Name
- School Address
- City
- Postal code
- Email
- Phone number

As *Children and young people's reading 2021* was designed to describe and summarize reading habits in each of the four target grades, it was important that the sampling frames gave comprehensive coverage of each national target population. However, as is often the case with sampling in educational studies (e.g. LaRoche et al., 2017, p. 3.6), the project chose to exclude a subset of students from the target populations because of social and operational factors (Pettersson et al., 2022). Students were excluded based on the following criteria:

<u>School Level Exclusions</u>

- Very small schools (less than four students on a relevant target grade)
- Schools only providing instruction to students in the student-level exclusion categories listed below (i.e. special needs students)

<u>Student Level Exclusions</u>

- Students with functional disabilities: students who had physical disabilities that meant they could not answer the survey

- Students with intellectual disabilities: students who were evaluated by teachers to have intellectual disabilities beyond poor academic performance. It also covered children who were either mentally or emotionally unable to follow the general instruction. However, schools were informed that they were not allowed to exclude children based solely on poor academic performance
- Non-native language speakers: students who typically had received less than one year of instruction in the native language at the time of the study
- Parents rejected participation: students whose parents refused their children's participation in the study

In addition, a number of schools were excluded because of the sampling design. Schools were sampled with an overlap in each sampling plan, i.e. schools were sampled to participate with students in two target grades (either 5th and 6th or 7th and 8th grade), thereby making it necessary for schools to have students enrolled in both grades to qualify. For instance, if a school for some reason had students in the fifth grade, but not in the sixth, the school was excluded from the sampling frame. Clearly, in this example, this would lower the population coverage of students in the fifth grade.

S1-2 Table documents population coverage and exclusion for each target group. It shows the number of students in each national target population and the number of students excluded at both the school level and within the school (i.e. student-level exclusion). As shown in the table, national coverage of the four target populations was in the range of 95-97 %.

S1-2 Table. Coverage and exclusion in the four target populations

	5th grade		6th grade		7th grade		8th grade	
	Schools	Students	Schools	Students	Schools	Students	Schools	Students
Total population	1873	68806	1883	67880	1659	69329	1747	68222
School-level exclusion	274	2126	284	2224	340	2467	428	3421
1. Treatment centers for students with psychosocial difficulties	81	301	84	361	104	413	118	527
2. Schools for functionally disabled students	117	1071	119	1093	117	1125	116	1058
3. Small schools	35	101	47	134	63	168	81	224
4. German/English/Waldorf schools	10	137	8	123	8	117	4	98
5. Given sampling design	31	516	26	513	48	644	109	1514
Student-level exclusion		17		19		10		16
Total exclusion	274	2143	284	2243	340	2477	428	3437
Expected coverage	1599	66663	1599	65637	1319	66852	1319	64785
Percent coverage	85%	97%	85%	97%	80%	96%	76%	95%

Notes. Information about the population size and the statistics related to school-level exclusion is based on data from the school year 2018/2019. It was received from the Danish Departmental Agency for IT and learning [STIL; Styrelsen for IT og Læring]. Student-level exclusion is based on information from participating schools during the data collection (Pettersson et al., 2022).

Sampling design

The basic sample design used is a stratified two-stage cluster sample design with a sample of schools drawn as a first stage and one intact class of students selected from each target grade as a second stage. The selection of schools was split into two independent sampling plans. In one sampling plan, schools were selected to participate with a class in the fifth and sixth grade from a list of schools with students enrolled in the two target grades. In the other sampling plan, schools were sampled to participate with a class in the seventh and the eighth grade from a list of schools with students enrolled in both the seventh and eighth grade. Sampling was divided into two sampling plans instead of one because it would be resource-intensive for schools to participate with a class in four different grades. Also, splitting the four grade levels into two sampling plans had an additional benefit, because a substantial number of schools in Denmark cover the 5th and the 6th grade (also known as "middle school", *mellemtrinnet in Danish*) but not the 7th and 8th grade (equivalent to the first years of "lower secondary", *udskolingen in Danish*). Thus, having two sampling plans instead of one increased the coverage of each target population.

Stratified two-stage cluster design

The stratified two-stage cluster sample design used mirrored the Danish sampling design in PIRLS 2016 (Mejding et al., 2017)[1]. Both sampling plans were executed as follows (Pettersson et al., 2022).

First stage: In the first sampling stage, schools were selected based on a sampling frame. Before selection, schools were divided into two groups based on school type (i.e. public or private) using explicit stratification. Within each explicit stratum, the schools were then selected using a systematic random sampling with probabilities proportional to size (PPS). The PPS technique gives larger schools, i.e. those with more students, a higher chance of being selected than smaller schools. But this difference in selection probabilities among larger and smaller schools is counterbalanced in the second sampling stage, where a fixed number of classes are selected with equal probability from each sampled school[2]. In addition, two replacement schools were selected for each sampled school. If a sampled school refused to participate, replacement schools were used to limit a loss in sample size.

Second stage: Within each participating school, one intact class of students were selected from each target grade[3]. Every sampled school provided a list of classes in the target grade that they were chosen to participate with. For instance, schools sampled to participate with a class in the fifth and the sixth grade provided two lists: one with all classes in the fifth grade and another with all classes in the sixth grade. Using these lists, a class from each grade was selected using simple random sampling.

Considerations about sample sizes

Children and young people's reading 2021 aimed to achieve reasonably small standard errors for survey estimates. The student sample for each grade level should ensure a level of precision defined by confidence intervals of ±3.5 percentage points for percentages (Pettersson et al., 2022). To meet

[1] The only difference is that *Children and young people's reading 2021* during the second stage sampled classes in two different target grades instead of in one target grade. Stratification variables used were identical.
[2] Under ideal conditions with a stratified PPS two-stage cluster sample, that is, with no non-response or disproportional sampling of subpopulations, this method leads to "self-weighted" samples where all units sampled in the last stage of sampling have similar selection probabilities (LaRoche et al., 2017).
[3] In a few instances, schools were allowed to participate with all classes in a target grade.

this precision requirement, *Children and young people's reading 2021* required an effective sample of 800 students on each target grade, i.e. if students were sampled with simple random sampling.

However, when cluster sampling is used, it is necessary to take account of the effect of intra-cluster correlation (i.e. ICC, the strength of correlation within clusters) on sample size calculations. This effect is known as the design effect (DEFF), a correction factor that is used to adjust the required sample size for the cluster sampling. To get an estimate of the design effect, the project used Danish data from PIRLS 2016, which had a similar stratified two-stage cluster design[4], and calculated the design effect for one of this project's main outcome, reading enjoyment (Pettersson et al., 2022). Subsequently, the design effect was used to adjust and estimate the number of schools needed to participate, and the student sample, albeit the design effect naturally varies on different outcomes depending on the ICC for the outcome.

S1-3 Table displays the calculation of sample size requirement, and it includes an adjustment for expected non-response on both the school- and student-level. It shows that precision requirements would be met with a school sample of 120 schools and a student sample of 1920 students. However, when including expected school (and student) non-response, at least 150 schools had to be sampled.

S1-3 Table. Sample size requirement for a target grade

Trait	Indicator	Number
Effective sample size	a	800
Design effect	b	2.4
Actual sample size	c = a x b	1920
Students per class	d	18.7
Minimum student response	e	0.85
Average response per class	f = d x e	15.9
Number of schools required	g = c/f	120.8
Minimum school non-response	h = 80%	0.80
Sample size (schools)	i = g/h	~ 150

Data collection and participation

The data collection included multiple stages, before students reached the survey response stage:

1. **stage:** Invitation and recruitment of schools to participate. Schools were invited by email. If schools did not respond, the headmaster was contacted by telephone. Recruitment of schools to participate occurred in spring 2021.[5]

2. **stage:** Collecting information on respondents from school administrations, i.e. name, gender, birth date, and email. Schools were obliged to send administrative information on participating children. The collection of information happened in August and September 2021.

[4] Similar to the Danish sample design in PIRLS 2016, *Children and young people's reading 2021* used explicit stratification in terms of school type and a two-stage PPS sampling method (Mejding et al., 2017).

[5] In each municipality (n=98), the head of the school administration was briefed about the study before schools were invited to participate.

3. **stage:** Collecting survey responses. Responses were collected between September 15th and November 17th, 2021.

Students completed the online survey in a classroom setting. The survey was made available through personalized links that were sent out to students' emails through the survey tool SurveyXact. In each school, a school coordinator (e.g., a teacher or a secretary) were present during the survey response session. School coordinators were briefed about the response session by the project team. Their responsibilities included: introducing the survey to the children, logging in, leading students through the survey, and making sure that students who fell ill were given a later chance to participate.

To ensure representativeness in a study, minimizing non-response bias is essential. As shown in S1-4 Table, the study had approximately 2000 students participating at each grade level and achieved noteworthy participation rates: 70-75% for schools and around 85% for students in each target grade. Altogether, the data collection results in a total sample of 8171 students from the 5th to 8th grade participating from 199 unique schools[6].

S1-4 Table. Participation across target grade

	5th grade	6th grade	7th grade	8th grade	Total
Schools	115 (75 %)	114 (74 %)	105 (70 %)	106 (70 %)	199
Students	2119 (85 %)	2118 (85 %)	1976 (85 %)	1958 (84 %)	8171

Note. Numbers show participating entities and the participation rate is in parentheses.

S1-5 Table presents the demographic composition of the total sample and population. The sample consisted of an equal split between boys and girls, as did the population. The distribution of grade was roughly divided in four equally sized groups in both the sample and population. Public school attendance accounted for 78.8% of the sampled children, slightly lower than that of the total population at 80.2%. Sample proportions across small, medium, and large schools were similar to those found in the overall population (33.9 vs 33.4%, 35.0 vs 33.4 %, and 31.0 vs 33.2%). In terms of home language spoken by students surveyed, Danish was predominant at a rate of 75% among the sampled, unfortunately we do not know this information in the population.

[6] 199 unique schools participated across the four target grades. A number of these, 25, were sampled by chance to participate with a class in the 5th and 6th grade along with a class in the 7th and 8th grade.

S1-5 Table. Characteristics of total sample of students in 5th to 8th grade

	Total sample<br>% (n)	Total population<br>%
Gender		
Girl	50.1 (4094)	49.3
Boy	49.9 (4077)	50.7
Grade		
5th	25.9 (2119)	24.6
6th	25.9 (2118)	25.4
7th	24.2 (1976)	24.9
8th	24.0 (1958)	25.1
School type		
Public	78.8 (6438)	80.4
Private	21.2 (1733)	19.6
School size		
Small (≤ 438 students)	33.9 (2772)	33.4
Medium	35.0 (2862)	33.4
Large (> 655 students)	31.0 (2537)	33.2
Language at home		
Danish	75.0 (6127)	NA
Other	25.0 (2044)	NA
Total	100.0 (8141)	100.0

Notes. Information about the population is based on data about all Danish students from the 5th to the 8th grade in the school year 2020/2021. It was collected from the Danish educational data warehouse [Uddannelsesstatistik]. Categories on school size are defined based on population terciles.

In the following, we present descriptive statistics on the student responses in terms of response time and dates conditional on grade. S1-6 Table shows the number of completed responses, mean response time, and median response time by target grade. Fig S1-1 shows the number of responses conditional on response date, while Fig S1-2 presents the distribution of survey response time (in minutes) with the red dotted line highlighting the mean response time.

S1-6 Table. Completed responses and response time by grade

	Complete responses	Mean response time (minutes)	Median response time (minutes)
5th grade	2219	26.7	24.9
6th grade	2118	22.6	20.9
7th grade	1976	18.9	17.6
8th grade	1958	16.3	15.3

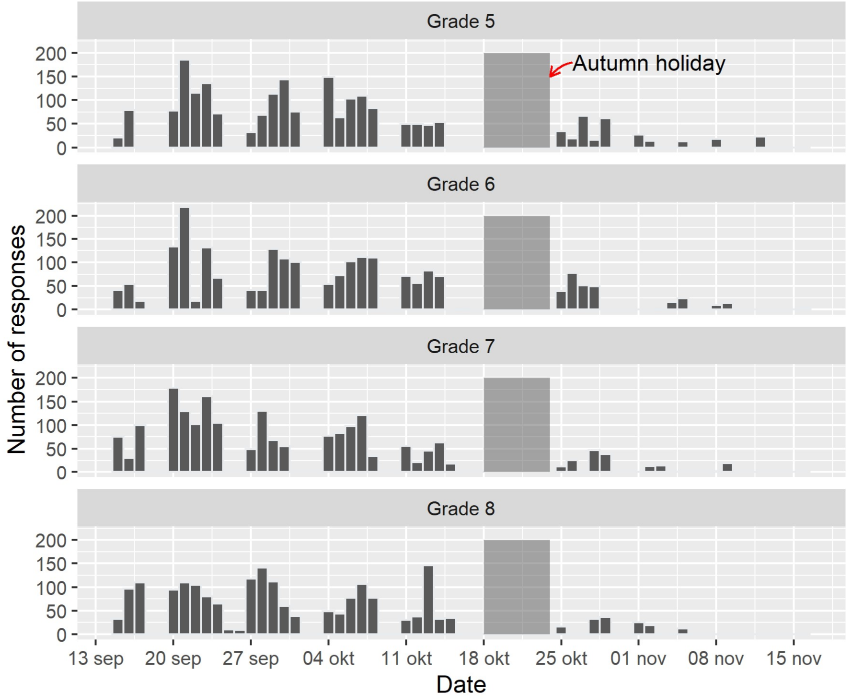

S1-1 Fig. Number of responses by date

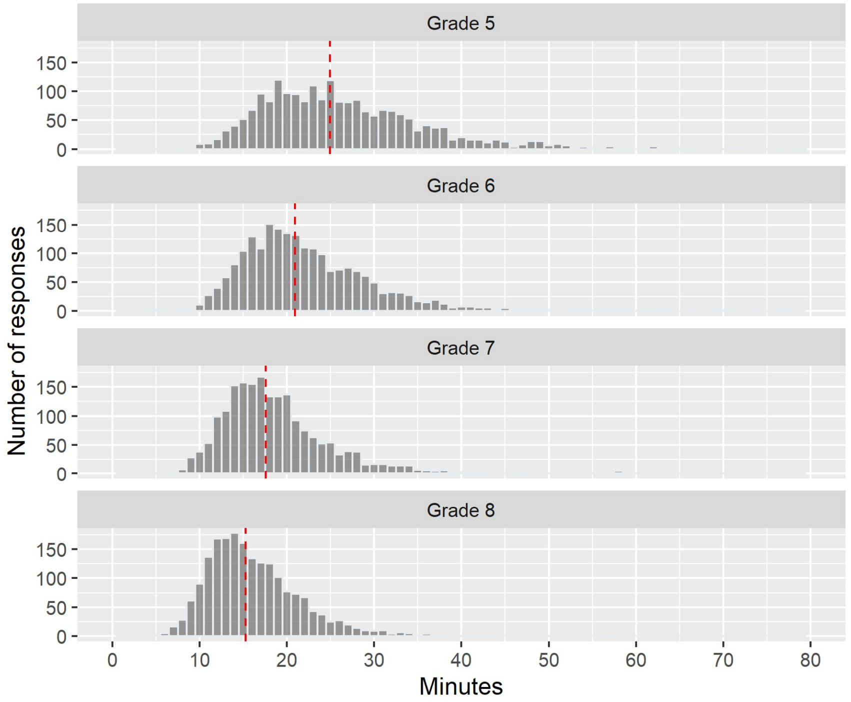

S1-2 Fig. Distribution of response time

Comparing student samples and target population in each grade

This section uses the information available about the sample and the population to explore if the sample of each target grade was demographically similar to the population. All variables used here (gender, school type and school size) are based on administrative information received either from schools or from the Danish educational data warehouse [Uddannelsesstatistik] (school year 2020/2021), available from the Danish Ministry of children and education at https://uddannelsesstatistik.dk/Pages/main.aspx.

Four tables are supplied in this section, i.e. one for each target grade. Each table presents a comparison between participating students (sample) and the population on the previously mentioned characteristics. The first column (1) shows the distribution in the sample calculated with design-weighted data[7]. Next, follows the distribution in the population (column 2). Column (3) shows the mean difference between these two groups, and column (4) the standard error of the difference. Standard errors are derived using the Jackknife repeated replication method for variance estimation (Foy 2013).[8]

---

[7] The project *Children and young people's reading 2021* calculated sampling weights for each grade in order to make accurate estimates of population traits for each of the four target groups. Under ideal conditions with a stratified PPS two-stage cluster sample, that is, with no non-response and no disproportional sampling of subpopulations, this design would lead to "self-weighted" samples where all units sampled in the last stage of sampling have similar selection probabilities (LaRoche et al., 2017). However, in reality, and also in this data collection, there was some non-response. Consequently, sampling weights were calculated to account for varying selection probabilities.

Sampling weights were computed separately for each populations, corresponding to the target grade that each sample were designed to represent. They were calculated using the exact same procedure as in PIRLS 2016 (LaRoche et al., 2017, pp. 3.18-3.22) because the sampling design mirrored the stratified two-stage cluster design used in PIRLS 2016. The final student sampling weight accounted for selection at three levels: school, class, and student; each level had a base weight, i.e. the inverse of the selection probability, and an adjustment for nonparticipation. Additional information on calculating sampling weights is available upon request from the corresponding authors.

[8] To estimate the sampling variance, we used the Jackknife Repeated Replication method for variance estimation. It is computationally straightforward, provides unbiased estimates of the sampling variance and appears to be the most-used approach in educational surveys when exploiting a two-stage cluster sample design (Foy & LaRoche, 2017; Schulz, 2020). In essence, the JRR technique works by grouping the PSUs (schools) two-and-two into Jackknife sampling *zones* in accordance with the stratification in the sample design, before repeated samples are drawn from each sampling zone, compared in terms of variability against the original full sample, and then used to obtain an estimate of the sampling variance. The JJR technique applied followed the procedure used in PIRLS 2011 (Foy, 2013). In a first step, schools were paired into sampling zones. The sampling zones were constructed within each explicit stratum, and we paired schools adjacent to each other in the sorted sample frame into one sampling zone. If an explicit stratum had an odd number of schools, the students in the remaining school were randomly divided into two quasi schools, thus forming an extra sampling zone. We constructed 58 sampling zones in the sample for students in the 5th grade (among 115 schools), 58 sampling zones in the sample for 6th grade (114 schools), 53 sampling zones in the sample for 7th grade (105 schools), and 54 sampling zones in the sample for 8th grade (106 schools). In the next step, we assigned a multiplication value to each school. Within each of the sampling zones, we randomly assigned one school a multiplication value of 2 and the other school a value of 0. For each of the sampling zones, we computed a replicate weight. In short, this means that one of the paired schools had a contribution of 0, the second a double contribution, and all other schools remained the same. The replicate weight was derived by multiplying the final student weight with the jackknife's multiplication value for the respective sampling zone while keeping student weights for all other zones unchanged. Thus, for instance, in the sample for students in the 5th grade, we ended up with 58 subsamples that generated 58 unique replicate sampling weights. The replicate sampling weights were then used to estimate the statistic of interest 58 times. The variation across these jackknife estimates provided an estimate of the sampling variance. For a more comprehensive description of the JRR technique applied, please see Foy 2013.

S1-7 Table. Differences in observed characteristics in the sample of students from the 5th grade

	Sample	Population	Difference	S.E
	%	%		
Gender				
Girl	50.8	49.2	1.6	0.9
Boy	49.2	50.8	-1.6	0.9
School type				
Public	81.0	81.9	0.9	0.7
Private	19.0	18.1	-0.9	0.7
School size				
Small	34.1	33.3	0.7	2.5
Medium	34.3	33.3	1.0	4.0
Large	31.6	33.4	-1.8	3.3

Note: Categories on school size are defined based on target population terciles.

S1-8 Table. Differences in observed characteristics in the sample of students from the 6th grade

	Sample	Population	Difference	S.E
	%	%		
Gender				
Girl	50.2	49.3	0.9	1.0
Boy	49.8	50.7	-0.9	1.0
School type				
Public	80.1	81.5	-1.4	0.7
Private	20.0	18.5	1.4	0.7
School size				
Small	33.8	33.3	0.4	2.9
Medium	35.5	33.3	2.2	4.3
Large	30.7	33.4	-2.6	3.3

Note: Categories on school size are defined based on target population terciles.

S1-9 Table. Differences in observed characteristics in the sample of students from the 7th grade

	Sample	Population	Difference	S.E
	%	%		
Gender				
Girl	50.4	49.5	0.9	1.2
Boy	49.6	50.5	-0.9	1.2
School type				
Public	78.4	79.0	-0.7	1.1
Private	21.6	21.0	0.7	1.1
School size				
Small	37.8	33.5	4.3	3.5
Medium	34.5	33.2	1.3	4.2
Large	27.6	33.3	-5.7	3.5

Note: Categories on school size are defined based on target population terciles.

S1-10 Table. Differences in observed characteristics in the sample of students from the 8th grade

	Sample	Population	Difference	S.E
	%	%		
Gender				
Girl	49.7	49.3	0.4	1.1
Boy	50.3	50.7	-0.4	1.1
School type				
Public	77.7	79.0	-1.2	1.2
Private	22.3	21.0	1.2	1.2
School size				
Small	36.9	33.3	3.6	3.5
Medium	35.7	33.3	2.5	4.1
Large	27.3	33.4	-6.1	3.4

Note: Categories on school size are defined based on target population terciles.

References

Foy, P., & LaRoche, S. (2017). Estimating Standard Errors in the PIRLS 2016 Results. In M. O. Martin, I. V. S. Mullis, & M. Hooper (Eds.), *Methods and Procedures in PIRLS 2016* (pp. 4.1–22). Chestnut Hill, MA: TIMSS & PIRLS International Study Center, Boston College.

Foy, P. (2013). Estimating standard errors for the TIMSS and PIRLS 2011 achievement scales. In M. O. Martin & I. V. S Mullis (Eds.), *Methods and Procedures in TIMSS and PIRLS 2011.* Chestnut Hill, MA: TIMSS & PIRLS International Study Center, Boston College.

Hansen, S. R., Hansen, T. I., & Pettersson, M. (2022). *Børn og unges læsning 2021*. Aarhus Universitetsforlag.

LaRoche, S., Joncas, M., & Foy, P. (2017). Sample Design in PIRLS 2016. In M. O. Martin, I. V. S. Mullis, & M. Hooper (Eds.), *Methods and Procedures in PIRLS 2016* (pp. 3.1–4). Chestnut Hill, MA: TIMSS & PIRLS International Study Center, Boston College.

Mejding, J., Neubert, K., & Larsen, R. (2017). *PIRLS 2016: en international undersøgelse om læsekompetence i 3. og 4. klasse: rapport*. Aarhus Universitetsforlag.

Pettersson, M., Erkmen, J., & Puck, M. R. (2022). *Børn og unges læsning 2021: Teknisk rapport.* UCL Erhvervsakademi og Professionshøjskole.

Schulz, W. (2020). The Reporting of ICILS Results. In J. Fraillon, J. Ainley, W. Schulz, T. Friedman, & D. Duckworth (Eds.), *ICILS 2018 Technical Report* (pp. 221-234). International Association for the Evaluation of Educational Achievement.
