# Supplementary material for: Measuring child and adolescent well-being in Denmark: Validation and norming of the Danish KIDSCREEN-10 child/adolescent version in a national representative sample of school pupils in grades five through eight
Source: PLoS One. 2023 Sep 8;18(9):e0291420. doi: 10.1371/journal.pone.0291420 (PMC10490965; doi:10.1371/journal.pone.0291420)
Supplement: S2 File — (PDF) [file pone.0291420.s003.pdf]

S2 File. Documentation of the initial evidence against fit the Rasch model, and evidence against fit for the last analysis attempted on the full sample

Initial evidence against the Rasch model for the full sample

Summary of global test results (homogenity across scoregroups and invariance across exogenous variables)

Delta will be reported if estimation did not converge.

	CLR	df	p	delta
scoregroups	746.3	39	0.000	
K: Language	343.7	39	0.000	
L: School	95.1	39	0.000	
M: Sex	1179.8	39	0.000	
N: Grade	620.3	117	0.000	

Critical levels adjusted by the Benjamini-Hochberg procedure:

FDR = 0.05	reject if p<= 0.0500
FDR = 0.01	reject if p<= 0.0100
FDR = 0.001	reject if p<= 0.0010

Tests of item fit

Conditional outfits and infits

		Outfit			Infit		
Item		observed	sd	p	observed	sd	p
A -	Kid1	0.819	0.017	0.00000***	0.811	0.016	0.00000***
B -	Kid2	0.980	0.015	0.19994	0.964	0.016	0.02093*
C -	Kid3	1.078	0.015	0.00000***	1.059	0.015	0.00012***
D -	Kid4	1.000	0.017	0.99636	0.996	0.016	0.79320
E -	Kid5	1.056	0.015	0.00024***	1.048	0.015	0.00199**
F -	Kid6	1.022	0.016	0.16744	1.014	0.016	0.37784
G -	Kid7	1.316	0.027	0.00000***	1.207	0.022	0.00000***
H -	Kid8	0.897	0.019	0.00000***	0.934	0.018	0.00025***
I -	Kid9	1.096	0.016	0.00000***	1.097	0.016	0.00000***
J -	Kid10	0.955	0.016	0.00523**	0.953	0.017	0.00498**

Item restscore association

		Item-restscore gamma			
Item		observed	expected	sd	p
A -	Kid1	0.642	0.523	0.008	0.00000***
B -	Kid2	0.533	0.505	0.008	0.00111**
C -	Kid3	0.477	0.506	0.008	0.00053***
D -	Kid4	0.540	0.507	0.008	0.00012***
E -	Kid5	0.492	0.511	0.008	0.01907*
F -	Kid6	0.513	0.505	0.008	0.38438
G -	Kid7	0.458	0.501	0.010	0.00002***

```
H -      Kid8    0.549    0.486    0.010  0.00000***
I -      Kid9    0.439    0.502    0.009  0.00000***
J -     Kid10    0.524    0.495    0.009  0.00117**
-----------------------------------------------
```

```
Critical levels adjusted by the Benjamini-Hochberg procedure:
    * < 5 % FDR, ** < 1 % FDR, *** = FDR < 0.1 % FDR

Benjamini-Hochberg limits for all outfits, infits and gamma coefficients

FDR = 5 %. Limit =  0.04000
FDR = 1 %. Limit =  0.00733
```

Evidence against fit for the last analysis attempted on the full sample

Confirmatory test for presence of local dependence in model

```
Standardized gamma coefficients

AB: Kid1 & Kid2         lr = 1178.52 df = 16 p = 0.0000 Gamma =  0.40
CD: Kid3 & Kid4         lr = 1433.51 df = 16 p = 0.0000 Gamma =  0.49
DH: Kid4 & Kid8         lr = 646.85  df = 16 p = 0.0000 Gamma =  0.29
EF: Kid5 & Kid6         lr = 684.95  df = 16 p = 0.0000 Gamma =  0.27
IJ: Kid9 & Kid10        lr = 1125.94 df = 16 p = 0.0000 Gamma =  0.43
```

Confirmatory test for presence of DIF in model

```
BN: Kid2 & Grade        lr =  60.28 df = 12 p = 0.0000 gamma = -0.11
CM: Kid3 & Sex          lr = 361.16 df =  4 p = 0.0000 gamma = -0.45
CN: Kid3 & Grade        lr =  93.58 df = 12 p = 0.0000 gamma =  0.13
EK: Kid5 & Language     lr =  41.07 df =  4 p = 0.0000 gamma =  0.11
EM: Kid5 & Sex          lr =  61.03 df =  4 p = 0.0000 gamma = -0.11
GM: Kid7 & Sex          lr =  75.36 df =  4 p = 0.0000 gamma =  0.17
IK: Kid9 & Language     lr =  31.35 df =  4 p = 0.0000 gamma =  0.12
IM: Kid9 & Sex          lr =  66.00 df =  4 p = 0.0000 gamma =  0.18
IN: Kid9 & Grade        lr = 148.21 df = 12 p = 0.0000 gamma = -0.16
JL: Kid10 & School      lr =  29.40 df =  4 p = 0.0000 gamma =  0.13
JM: Kid10 & Sex         lr =  40.52 df =  4 p = 0.0000 gamma =  0.15
```

Summary of global test results (homogenity across scoregroups and invariance across exogenous variables)

```
             CLR   df   p        delta
--------------------------------------
scoregroups  913.0 187 0.000
K: Language  414.8 171 0.000
L:   School  183.1 179 0.402
M:      Sex  336.1 147 0.000
N:    Grade  648.9 417 0.000

Critical levels adjusted by the Benjamini-Hochberg procedure:

 FDR = 0.05         reject if p<=  0.0500
 FDR = 0.01         reject if p<=  0.0100
 FDR = 0.001        reject if p<=  0.0010
```

Tests of item fit

Item		Outfit observed	sd	p	Infit observed	sd	p
A -	Kid1	0.820	0.018	0.00000***	0.816	0.016	0.00000***
B -	Kid2	1.011	0.016	0.48858	0.996	0.016	0.82442
C -	Kid3	1.137	0.015	0.00000***	1.131	0.016	0.00000***
D -	Kid4	1.190	0.020	0.00000***	1.153	0.018	0.00000***
E -	Kid5	1.026	0.016	0.09829	1.020	0.016	0.19752
F -	Kid6	1.000	0.017	0.99993	0.994	0.017	0.72184
G -	Kid7	1.025	0.025	0.33457	0.993	0.023	0.76177
H -	Kid8	0.899	0.021	0.00000***	0.939	0.020	0.00212**
I -	Kid9	1.076	0.017	0.00000***	1.070	0.017	0.00003***
J -	Kid10	0.950	0.017	0.00294**	0.950	0.017	0.00391**

Item restscore association

Item		Item-restscore gamma observed	expected	sd	p
A -	Kid1	0.642	0.532	0.008	0.00000***
B -	Kid2	0.533	0.527	0.008	0.50743
C -	Kid3	0.477	0.541	0.008	0.00000***
D -	Kid4	0.540	0.585	0.008	0.00000***
E -	Kid5	0.492	0.497	0.008	0.61403
F -	Kid6	0.513	0.495	0.009	0.03614*
G -	Kid7	0.458	0.392	0.011	0.00000***
H -	Kid8	0.549	0.496	0.010	0.00000***
I -	Kid9	0.439	0.486	0.009	0.00000***
J -	Kid10	0.524	0.494	0.009	0.00074**

Critical levels adjusted by the Benjamini-Hochberg procedure:
* < 5 % FDR, ** < 1 % FDR, *** = FDR < 0.1 % FDR

Component-restscore gamma coefficients

Component	Gamma observed	expected	sd	p
AB	0.566	0.490	0.007	0.0000
CDH	0.467	0.502	0.007	0.0000
EF	0.493	0.485	0.007	0.2848
IJ	0.443	0.451	0.008	0.3160

Benjamini-Hochberg limits for all outfits, infits and gamma coefficients

FDR = 5 %. Limit = 0.03088
FDR = 1 %. Limit = 0.00618
