# Supplementary material for: Measuring child and adolescent well-being in Denmark: Validation and norming of the Danish KIDSCREEN-10 child/adolescent version in a national representative sample of school pupils in grades five through eight
Source: PLoS One. 2023 Sep 8;18(9):e0291420. doi: 10.1371/journal.pone.0291420 (PMC10490965; doi:10.1371/journal.pone.0291420)
Supplement: S3 File — (PDF) [file pone.0291420.s004.pdf]

## S3 File. Additional results for each of the models resulting from analyses of the random samples

In this file, we provide the detailed results showing evidence against or for fit to each of the models. Any degree of evidence against fit of the response set to a given model is marked in red.

**Prior to model 1**

Test of fit to the Rasch model.

Summary of global test results (homogenity across scoregroups and invariance across exogenous variables)

Delta will be reported if estimation did not converge.

| | CLR | df | p |
|---|---|---|---|
| scoregroups | 44.8 | 38 | 0.207 |
| K: Language | 34.8 | 38 | 0.619 |
| L: School | 35.2 | 38 | 0.599 |
| M: Sex | 84.5 | 38 | 0.000 |
| N: Grade | 102.7 | 114 | 0.767 |

Critical levels adjusted by the Benjamini-Hochberg procedure:

| | |
|---|---|
| FDR = 0.05 | reject if p<= 0.0100 |
| FDR = 0.01 | reject if p<= 0.0020 |
| FDR = 0.001 | reject if p<= 0.0002 |

Tests of item fit

Conditional outfits and infits

| Item | | Outfit observed | sd | p | Infit observed | sd | p |
|---|---|---|---|---|---|---|---|
| A - | Kid1 | 0.736 | 0.100 | 0.00846 | 0.769 | 0.095 | 0.01445 |
| B - | Kid2 | 0.992 | 0.094 | 0.93242 | 0.994 | 0.096 | 0.95398 |
| C - | Kid3 | 1.230 | 0.093 | 0.01312 | 1.228 | 0.090 | 0.01159 |
| D - | Kid4 | 0.886 | 0.105 | 0.27700 | 0.970 | 0.094 | 0.74944 |
| E - | Kid5 | 1.072 | 0.094 | 0.44159 | 1.065 | 0.093 | 0.48870 |
| F - | Kid6 | 1.135 | 0.100 | 0.17629 | 1.066 | 0.100 | 0.50652 |
| G - | Kid7 | 1.087 | 0.169 | 0.60597 | 1.054 | 0.133 | 0.68438 |
| H - | Kid8 | 0.785 | 0.126 | 0.08980 | 0.854 | 0.115 | 0.20494 |
| I - | Kid9 | 1.124 | 0.104 | 0.23370 | 1.065 | 0.102 | 0.52701 |
| J - | Kid10 | 1.022 | 0.100 | 0.82903 | 1.005 | 0.103 | 0.95971 |

Item restscore association

| Item | | Item-restscore gamma observed | expected | sd | p |
|---|---|---|---|---|---|
| A - | Kid1 | 0.652 | 0.513 | 0.050 | 0.00558 |
| B - | Kid2 | 0.514 | 0.495 | 0.052 | 0.70871 |
| C - | Kid3 | 0.393 | 0.503 | 0.050 | 0.02725 |
| D - | Kid4 | 0.570 | 0.493 | 0.053 | 0.14519 |
| E - | Kid5 | 0.458 | 0.498 | 0.051 | 0.42692 |
| F - | Kid6 | 0.480 | 0.496 | 0.052 | 0.75152 |

```
G -      Kid7     0.533    0.484    0.063  0.43659
H -      Kid8     0.597    0.467    0.061  0.03270
I -      Kid9     0.453    0.496    0.056  0.44184
J -     Kid10     0.473    0.488    0.055  0.79251
------------------------------------------------

Critical levels adjusted by the Benjamini-Hochberg procedure:
    * < 5 % FDR, ** < 1 % FDR, *** = FDR < 0.1 % FDR

Benjamini-Hochberg limits for all outfits, infits and gamma coefficients

FDR = 5 %. Limit =  0.00167
FDR = 1 %. Limit =  0.00033
```

Tests of local independence

```
C & D:   lr =   52.18  df =  12  p = 0.0000  WPG gamma =   0.68
```

Tests of missing DIF

```
C & M:   lr =   28.87  df =   4  p = 0.0000 gamma =  -0.56
```

**Model 1 (n=214)**
Test of fit to the GLLRM with DIF DM shown in Figure AA.

Confirmatory test for presence of DIF in model

Standardized gamma coefficients

DM: Kid4 & Sex lr = 13.96 df = 3 p = 0.0030 gamma = -0.50

Summary of global test results (homogenity across scoregroups and invariance across exogenous variables)

Delta will be reported if estimation did not converge

| | CLR | df | p |
|---|---|---|---|
| scoregroups | 36.6 | 38 | 0.534 |
| K: Language | 31.7 | 38 | 0.754 |
| L: School | 36.5 | 38 | 0.537 |
| M: Sex | 42.1 | 31 | 0.089 |
| N: Grade | 106.1 | 114 | 0.689 |

Tests of item fit

Conditional outfits and infits

| Item | | Outfit observed | sd | p | Infit observed | sd | p |
|---|---|---|---|---|---|---|---|
| A - | Kid1 | 0.776 | 0.099 | 0.02460 | 0.818 | 0.095 | 0.05394 |
| B - | Kid2 | 1.024 | 0.094 | 0.79405 | 1.022 | 0.096 | 0.81674 |
| D - | Kid4 | 1.095 | 0.105 | 0.36727 | 1.157 | 0.095 | 0.09889 |
| E - | Kid5 | 1.063 | 0.094 | 0.49972 | 1.058 | 0.094 | 0.53876 |
| F - | Kid6 | 1.128 | 0.100 | 0.19840 | 1.058 | 0.100 | 0.56155 |
| G - | Kid7 | 1.050 | 0.173 | 0.77130 | 1.077 | 0.133 | 0.56387 |
| H - | Kid8 | 0.803 | 0.128 | 0.12385 | 0.854 | 0.115 | 0.20262 |
| I - | Kid9 | 1.083 | 0.103 | 0.42260 | 1.045 | 0.102 | 0.65422 |
| J - | Kid10 | 1.025 | 0.099 | 0.80283 | 0.993 | 0.103 | 0.94384 |

Item restscore association

| Item | | Item-restscore gamma observed | expected | sd | p |
|---|---|---|---|---|---|
| A - | Kid1 | 0.637 | 0.528 | 0.049 | 0.02626 |
| B - | Kid2 | 0.508 | 0.511 | 0.051 | 0.95226 |
| D - | Kid4 | 0.488 | 0.515 | 0.052 | 0.60280 |
| E - | Kid5 | 0.476 | 0.514 | 0.050 | 0.44520 |
| F - | Kid6 | 0.491 | 0.513 | 0.051 | 0.66819 |
| G - | Kid7 | 0.550 | 0.502 | 0.062 | 0.43295 |
| H - | Kid8 | 0.614 | 0.486 | 0.060 | 0.03238 |
| I - | Kid9 | 0.478 | 0.511 | 0.055 | 0.54920 |
| J - | Kid10 | 0.487 | 0.504 | 0.054 | 0.75193 |

Critical levels adjusted by the Benjamini-Hochberg procedure:
* < 5 % FDR, ** < 1 % FDR, *** = FDR < 0.1 % FDR

Benjamini-Hochberg limits for all outfits, infits and gamma coefficients

FDR = 5 %. Limit = 0.00185
FDR = 1 %. Limit = 0.00037

## Effect of DIF

*Item scores have been recoded for analysis (1-5 → 0-4), and thus the sum scale score range was changed from 9-45 to 0-36.*

DIF equated scores

DIF sources:
M - Sex: 1 = Boy 2 = Girl

| score | M 1 | M 2 |
|---|---|---|
| 0 | 0 | 0 |
| 1 | 1.00 | 1.00 |
| 2 | 2.00 | 2.01 |
| 3 | 3.00 | 3.03 |
| 4 | 4.00 | 4.05 |
| 5 | 5.00 | 5.07 |
| 6 | 6.00 | 6.10 |
| 7 | 7.00 | 7.13 |
| 8 | 8.00 | 8.16 |
| 9 | 9.00 | 9.20 |
| 10 | 10.00 | 10.23 |
| 11 | 11.00 | 11.27 |
| 12 | 12.00 | 12.31 |
| 13 | 13.00 | 13.34 |
| 14 | 14.00 | 14.38 |
| 15 | 15.00 | 15.41 |
| 16 | 16.00 | 16.44 |
| 17 | 17.00 | 17.47 |
| 18 | 18.00 | 18.49 |
| 19 | 19.00 | 19.51 |
| 20 | 20.00 | 20.52 |
| 21 | 21.00 | 21.53 |
| 22 | 22.00 | 22.53 |
| 23 | 23.00 | 23.52 |
| 24 | 24.00 | 24.51 |
| 25 | 25.00 | 25.48 |
| 26 | 26.00 | 26.46 |
| 27 | 27.00 | 27.42 |
| 28 | 28.00 | 28.39 |
| 29 | 29.00 | 29.34 |
| 30 | 30.00 | 30.30 |
| 31 | 31.00 | 31.25 |
| 32 | 32.00 | 32.20 |
| 33 | 33.00 | 33.15 |
| 34 | 34.00 | 34.09 |
| 35 | 35.00 | 35.05 |
| 36 | 36 | 36 |

Observed score

| | | |
|---|---|---|
| n | 105 | 109 |
| Mean | 27.36 | 25.06 |
| s.d. | 4.58 | 5.36 |
| s.e. | 0.45 | 0.51 |

Equated score

| | | |
|---|---|---|
| Mean | 27.36 | 25.46 |
| s.d. | 4.58 | 5.27 |
| s.e. | 0.45 | 0.50 |

Test bias

| | | |
|---|---|---|
| Bias | 0.00 | -0.40 |
| stand. | 0.00 | -0.08 |

**Model 2 (n = 413)**
Test of fit to the GLLRM with DIF AM, DM and EM and locally dependent items AB, AJ, EF, DH and IJ shown in Figure BB.

Confirmatory test for presence of local dependence in model

```
Standardized gamma coefficients

AB: Kid1 & Kid2         lr =  67.98 df = 16 p = 0.0000 Gamma =  0.28
AJ: Kid1 & Kid10        lr =  38.00 df = 16 p = 0.0015 Gamma =  0.21
DH: Kid4 & Kid8         lr =  44.52 df = 16 p = 0.0002 Gamma =  0.27
EF: Kid5 & Kid6         lr =  47.77 df = 16 p = 0.0001 Gamma =  0.27
IJ: Kid9 & Kid10        lr =  50.81 df = 16 p = 0.0000 Gamma =  0.48
```

Confirmatory test for presence of DIF in model

```
Standardized gamma coefficients

AM: Kid1 & Sex          lr =  18.93 df =  4 p = 0.0008 gamma = -0.41
DM: Kid4 & Sex          lr =  52.03 df =  4 p = 0.0000 gamma = -0.63
EM: Kid5 & Sex          lr =  20.95 df =  4 p = 0.0003 gamma = -0.37
IN: Kid9 & Grade        lr =  25.82 df = 12 p = 0.0114 gamma = -0.12
```

Summary of global test results (homogenity across scoregroups and invariance across exogenous variables)

```
Delta will be reported if estimation did not converge.

             CLR   df   p
-----------------------------
scoregroups  142.0 125 0.142
K: Language  147.2 125 0.085
L:   School  122.2 125 0.554
M:      Sex  122.3 101 0.073
N:    Grade  374.7 330 0.045
```

Tests of item fit

```
Conditional outfits and infits

              Outfit                          Infit
Item          observed     sd       p       observed     sd       p
--------------------------------------------------------------------
A -     Kid1     0.951    0.087  0.57695        0.869    0.078  0.09389
B -     Kid2     1.004    0.075  0.95544        0.998    0.077  0.97610
D -     Kid4     1.333    0.080  0.00003***     1.188    0.078  0.01611     low
E -     Kid5     1.041    0.073  0.57344        1.021    0.075  0.77637
F -     Kid6     0.972    0.073  0.69950        0.994    0.076  0.93220
G -     Kid7     0.901    0.109  0.36536        0.980    0.093  0.83465
H -     Kid8     0.940    0.089  0.50035        0.923    0.088  0.38150
I -     Kid9     1.068    0.074  0.36089        1.065    0.076  0.39195
J -    Kid10     0.967    0.075  0.66260        0.978    0.075  0.76655
--------------------------------------------------------------------
```

Item restscore association

| Item | | Item-restscore gamma observed | expected | sd | p |
|---|---|---|---|---|---|
| A - | Kid1 | 0.665 | 0.592 | 0.034 | 0.03474 |
| B - | Kid2 | 0.560 | 0.528 | 0.038 | 0.40416 |
| D - | Kid4 | 0.429 | 0.495 | 0.039 | 0.09248 |
| E - | Kid5 | 0.501 | 0.518 | 0.037 | 0.63942 |
| F - | Kid6 | 0.527 | 0.504 | 0.040 | 0.56411 |
| G - | Kid7 | 0.485 | 0.436 | 0.048 | 0.30920 |
| H - | Kid8 | 0.543 | 0.490 | 0.043 | 0.22573 |
| I - | Kid9 | 0.514 | 0.542 | 0.038 | 0.46316 |
| J - | Kid10 | 0.627 | 0.594 | 0.037 | 0.37705 |

Critical levels adjusted by the Benjamini-Hochberg procedure:
* < 5 % FDR, ** < 1 % FDR, *** = FDR < 0.1 % FDR

Component-restscore gamma coefficients

| Component | Gamma observed | expected | sd | p |
|---|---|---|---|---|
| ABIJ | 0.545 | 0.531 | 0.030 | 0.6410 |
| DH | 0.469 | 0.488 | 0.035 | 0.6025 |
| EF | 0.498 | 0.499 | 0.033 | 0.9802 |

Benjamini-Hochberg limits for all outfits, infits and gamma coefficients

FDR = 5 %. Limit = 0.00167
FDR = 1 %. Limit = 0.00033

## Effect of DIF

*Item scores have been recoded for analysis (1-5 → 0-4), and thus the sum scale score range was changed from 9-45 to 0-36.*

DIF sources:
M - Sex: 1 = Boy 2 = Girl
N - Grade: 1 = 5 2 = 6 3 = 7 4 = 8

| score | MN 11 | MN 21 | MN 12 | MN 22 | MN 13 | MN 23 | MN 14 | MN 24 |
|---|---|---|---|---|---|---|---|---|
| 0 | 0 | 0 | 0 | 0 | 0 | 0 | 0 | 0 |
| 1 | 1.00 | 0.92 | 1.28 | 1.15 | 0.34 | 0.32 | 0.98 | 0.91 |
| 2 | 2.00 | 1.79 | 2.41 | 2.14 | 1.26 | 1.13 | 1.96 | 1.76 |
| 3 | 3.00 | 2.67 | 3.49 | 3.12 | 2.39 | 2.10 | 2.93 | 2.62 |
| 4 | 4.00 | 3.59 | 4.54 | 4.10 | 3.46 | 3.06 | 3.90 | 3.50 |
| 5 | 5.00 | 4.53 | 5.57 | 5.11 | 4.51 | 4.03 | 4.87 | 4.41 |
| 6 | 6.00 | 5.52 | 6.59 | 6.14 | 5.55 | 5.04 | 5.84 | 5.36 |
| 7 | 7.00 | 6.53 | 7.59 | 7.18 | 6.58 | 6.08 | 6.80 | 6.33 |
| 8 | 8.00 | 7.58 | 8.60 | 8.25 | 7.61 | 7.15 | 7.77 | 7.33 |
| 9 | 9.00 | 8.67 | 9.59 | 9.34 | 8.63 | 8.25 | 8.73 | 8.36 |
| 10 | 10.00 | 9.79 | 10.58 | 10.45 | 9.65 | 9.39 | 9.70 | 9.44 |
| 11 | 11.00 | 10.95 | 11.56 | 11.60 | 10.67 | 10.58 | 10.67 | 10.56 |
| 12 | 12.00 | 12.14 | 12.55 | 12.77 | 11.69 | 11.80 | 11.65 | 11.71 |

| | | | | | | | | |
|---|---|---|---|---|---|---|---|---|
| 13 | 13.00 | 13.36 | 13.53 | 13.96 | 12.72 | 13.05 | 12.63 | 12.90 |
| 14 | 14.00 | 14.58 | 14.51 | 15.15 | 13.74 | 14.31 | 13.62 | 14.12 |
| 15 | 15.00 | 15.79 | 15.49 | 16.32 | 14.77 | 15.58 | 14.62 | 15.35 |
| 16 | 16.00 | 16.98 | 16.47 | 17.47 | 15.81 | 16.83 | 15.62 | 16.57 |
| 17 | 17.00 | 18.14 | 17.45 | 18.59 | 16.85 | 18.06 | 16.63 | 17.77 |
| 18 | 18.00 | 19.28 | 18.43 | 19.69 | 17.89 | 19.26 | 17.65 | 18.97 |
| 19 | 19.00 | 20.40 | 19.40 | 20.77 | 18.94 | 20.45 | 18.69 | 20.15 |
| 20 | 20.00 | 21.53 | 20.37 | 21.85 | 20.00 | 21.65 | 19.73 | 21.35 |
| 21 | 21.00 | 22.67 | 21.34 | 22.94 | 21.07 | 22.86 | 20.79 | 22.57 |
| 22 | 22.00 | 23.83 | 22.29 | 24.05 | 22.13 | 24.09 | 21.85 | 23.81 |
| 23 | 23.00 | 25.01 | 23.25 | 25.18 | 23.19 | 25.32 | 22.92 | 25.07 |
| 24 | 24.00 | 26.18 | 24.21 | 26.29 | 24.24 | 26.50 | 23.99 | 26.31 |
| 25 | 25.00 | 27.29 | 25.16 | 27.35 | 25.28 | 27.60 | 25.05 | 27.46 |
| 26 | 26.00 | 28.31 | 26.11 | 28.33 | 26.30 | 28.58 | 26.11 | 28.50 |
| 27 | 27.00 | 29.23 | 27.07 | 29.22 | 27.31 | 29.46 | 27.16 | 29.43 |
| 28 | 28.00 | 30.05 | 28.03 | 30.03 | 28.30 | 30.25 | 28.20 | 30.25 |
| 29 | 29.00 | 30.80 | 28.99 | 30.77 | 29.27 | 30.96 | 29.23 | 31.01 |
| 30 | 30.00 | 31.51 | 29.97 | 31.46 | 30.24 | 31.64 | 30.26 | 31.72 |
| 31 | 31.00 | 32.19 | 30.94 | 32.14 | 31.20 | 32.31 | 31.29 | 32.42 |
| 32 | 32.00 | 32.89 | 31.93 | 32.83 | 32.16 | 32.98 | 32.32 | 33.12 |
| 33 | 33.00 | 33.60 | 32.92 | 33.54 | 33.12 | 33.67 | 33.33 | 33.84 |
| 34 | 34.00 | 34.35 | 33.93 | 34.30 | 34.08 | 34.40 | 34.30 | 34.57 |
| 35 | 35.00 | 35.15 | 34.96 | 35.12 | 35.04 | 35.18 | 35.18 | 35.29 |
| 36 | 36 | 36 | 36 | 36 | 36 | 36 | 36 | 36 |

Observed score

| | | | | | | | | |
|---|---|---|---|---|---|---|---|---|
| n | 50 | 62 | 50 | 45 | 48 | 53 | 54 | 51 |
| Mean | 26.46 | 27.26 | 28.62 | 24.36 | 26.40 | 24.49 | 26.22 | 22.73 |
| s.d. | 5.83 | 4.69 | 4.38 | 5.52 | 4.54 | 6.39 | 4.80 | 5.66 |
| s.e. | 0.82 | 0.60 | 0.62 | 0.82 | 0.65 | 0.88 | 0.65 | 0.79 |

Equated score

| | | | | | | | | |
|---|---|---|---|---|---|---|---|---|
| Mean | 26.46 | 28.83 | 28.67 | 26.11 | 26.60 | 26.17 | 26.28 | 24.36 |
| s.d. | 5.83 | 4.36 | 4.24 | 5.51 | 4.62 | 6.88 | 4.96 | 6.39 |
| s.e. | 0.82 | 0.55 | 0.60 | 0.82 | 0.67 | 0.94 | 0.68 | 0.89 |

Test bias

| | | | | | | | | |
|---|---|---|---|---|---|---|---|---|
| Bias | 0.00 | -1.58 | -0.05 | -1.75 | -0.20 | -1.68 | -0.06 | -1.63 |
| stand. | 0.00 | -0.36 | -0.01 | -0.32 | -0.04 | -0.24 | -0.01 | -0.26 |

**Model 3 (n=630)**

Test of fit to the GLLRM with DIF AM, DM and EM and locally dependent items AB, AJ, EF, DH and IJ shown in Figure CC.

Confirmatory test for presence of local dependence in model

```
Standardized gamma coefficients

AB: Kid1 & Kid2         lr = 131.60 df = 16 p = 0.0000 Gamma =  0.46
BJ: Kid2 & Kid10        lr =  49.79 df = 16 p = 0.0000 Gamma =  0.02
DH: Kid4 & Kid8         lr =  85.95 df = 16 p = 0.0000 Gamma =  0.47
EF: Kid5 & Kid6         lr =  79.04 df = 16 p = 0.0000 Gamma =  0.35
FJ: Kid6 & Kid10        lr =  40.71 df = 16 p = 0.0006 Gamma =  0.02
IJ: Kid9 & Kid10        lr = 109.02 df = 16 p = 0.0000 Gamma =  0.47
```

Confirmatory test for presence of DIF in model

```
Standardized gamma coefficients

DM: Kid4 & Sex          lr =  17.33 df =  4 p = 0.0017 gamma = -0.28
IN: Kid9 & Grade        lr =  25.51 df = 12 p = 0.0126 gamma = -0.14
```

Summary of global test results (homogenity across scoregroups and invariance across exogenous variables)

```
Delta will be reported if estimation did not converge

             CLR   df   p
----------------------------
scoregroups  160.1 132 0.048
K: Language  132.3 132 0.477
L:   School  135.1 132 0.408
M:      Sex  161.6 124 0.013
N:    Grade  398.1 348 0.033

Critical levels adjusted by the Benjamini-Hochberg procedure:

 FDR = 0.05         reject if p<=  0.0500
 FDR = 0.01         reject if p<=  0.0020
 FDR = 0.001        reject if p<=  0.0002
```

Tests of item fit

```
Conditional outfits and infits

               Outfit                          Infit
Item          observed     sd        p        observed     sd        p
-----------------------------------------------------------------------
A -     Kid1    0.866    0.068  0.04780        0.869    0.061  0.03315
B -     Kid2    1.049    0.059  0.40385        1.049    0.059  0.41017
D -     Kid4    1.174    0.061  0.00424*       1.119    0.060  0.04757
E -     Kid5    0.985    0.056  0.79392        0.992    0.057  0.89279
F -     Kid6    0.973    0.066  0.68112        0.947    0.065  0.42075
G -     Kid7    1.054    0.084  0.52053        1.008    0.079  0.91622
H -     Kid8    0.877    0.064  0.05534        0.921    0.063  0.21190
I -     Kid9    1.083    0.059  0.15911        1.074    0.061  0.22318
```

| | | | | | | | |
|---|---|---|---|---|---|---|---|
| J - | Kid10 | 1.033 | 0.073 | 0.65570 | 1.032 | 0.072 | 0.66126 |

Item restscore association

| Item | | Item-restscore gamma observed | expected | sd | p |
|---|---|---|---|---|---|
| A - | Kid1 | 0.638 | 0.567 | 0.027 | 0.00948 |
| B - | Kid2 | 0.584 | 0.575 | 0.028 | 0.76584 |
| D - | Kid4 | 0.482 | 0.523 | 0.029 | 0.16123 |
| E - | Kid5 | 0.529 | 0.521 | 0.029 | 0.77662 |
| F - | Kid6 | 0.577 | 0.546 | 0.029 | 0.29914 |
| G - | Kid7 | 0.477 | 0.408 | 0.040 | 0.08172 |
| H - | Kid8 | 0.576 | 0.523 | 0.033 | 0.10662 |
| I - | Kid9 | 0.461 | 0.528 | 0.030 | 0.02618 |
| J - | Kid10 | 0.539 | 0.573 | 0.031 | 0.27449 |

Critical levels adjusted by the Benjamini-Hochberg procedure:
 * < 5 % FDR, ** < 1 % FDR, *** = FDR < 0.1 % FDR

Component-restscore gamma coefficients

| Component | Gamma observed | expected | sd | p |
|---|---|---|---|---|
| ABEFIJ | 0.528 | 0.505 | 0.025 | 0.3564 |
| DH | 0.483 | 0.486 | 0.027 | 0.9140 |

Benjamini-Hochberg limits for all outfits, infits and gamma coefficients

FDR = 5 %. Limit = 0.00172
FDR = 1 %. Limit = 0.00034

## Effect of DIF

*Item scores have been recoded for analysis (1-5 → 0-4), and thus the sum scale score range was changed from 9-45 to 0-36.*

DIF sources:
M - Sex: 1 = Boy 2 = Girl
N - Grade: 1 = 5 2 = 6 3 = 7 4 = 8

| score | MN 11 | MN 21 | MN 12 | MN 22 | MN 13 | MN 23 | MN 14 | MN 24 |
|---|---|---|---|---|---|---|---|---|
| 0 | 0 | 0 | 0 | 0 | 0 | 0 | 0 | 0 |
| 1 | 1.00 | 1.21 | 1.28 | 1.24 | 1.30 | 1.25 | 1.33 | 1.27 |
| 2 | 2.00 | 1.87 | 2.08 | 1.94 | 2.11 | 1.96 | 2.20 | 2.03 |
| 3 | 3.00 | 2.78 | 3.11 | 2.89 | 3.15 | 2.92 | 3.27 | 3.03 |
| 4 | 4.00 | 3.75 | 4.13 | 3.87 | 4.14 | 3.89 | 4.29 | 4.03 |
| 5 | 5.00 | 4.74 | 5.14 | 4.88 | 5.12 | 4.87 | 5.28 | 5.02 |
| 6 | 6.00 | 5.74 | 6.15 | 5.89 | 6.08 | 5.84 | 6.23 | 5.99 |
| 7 | 7.00 | 6.75 | 7.14 | 6.90 | 7.04 | 6.81 | 7.16 | 6.94 |
| 8 | 8.00 | 7.77 | 8.13 | 7.91 | 7.99 | 7.77 | 8.08 | 7.88 |
| 9 | 9.00 | 8.80 | 9.12 | 8.93 | 8.94 | 8.74 | 9.00 | 8.82 |

| | | | | | | | | |
|---|---|---|---|---|---|---|---|---|
| 10 | 10.00 | 9.82 | 10.11 | 9.94 | 9.89 | 9.72 | 9.92 | 9.76 |
| 11 | 11.00 | 10.85 | 11.09 | 10.95 | 10.85 | 10.70 | 10.85 | 10.71 |
| 12 | 12.00 | 11.89 | 12.08 | 11.97 | 11.82 | 11.70 | 11.79 | 11.68 |
| 13 | 13.00 | 12.92 | 13.07 | 12.99 | 12.80 | 12.71 | 12.75 | 12.66 |
| 14 | 14.00 | 13.95 | 14.06 | 14.01 | 13.79 | 13.73 | 13.72 | 13.66 |
| 15 | 15.00 | 14.99 | 15.05 | 15.04 | 14.79 | 14.77 | 14.71 | 14.69 |
| 16 | 16.00 | 16.03 | 16.04 | 16.07 | 15.80 | 15.82 | 15.72 | 15.74 |
| 17 | 17.00 | 17.07 | 17.03 | 17.10 | 16.83 | 16.89 | 16.74 | 16.80 |
| 18 | 18.00 | 18.11 | 18.02 | 18.13 | 17.86 | 17.96 | 17.78 | 17.89 |
| 19 | 19.00 | 19.15 | 19.02 | 19.17 | 18.90 | 19.05 | 18.84 | 18.98 |
| 20 | 20.00 | 20.19 | 20.01 | 20.20 | 19.94 | 20.14 | 19.89 | 20.09 |
| 21 | 21.00 | 21.23 | 21.00 | 21.23 | 20.99 | 21.23 | 20.96 | 21.19 |
| 22 | 22.00 | 22.26 | 21.99 | 22.25 | 22.04 | 22.31 | 22.02 | 22.29 |
| 23 | 23.00 | 23.29 | 22.98 | 23.27 | 23.08 | 23.39 | 23.07 | 23.38 |
| 24 | 24.00 | 24.32 | 23.97 | 24.28 | 24.12 | 24.46 | 24.12 | 24.46 |
| 25 | 25.00 | 25.34 | 24.95 | 25.29 | 25.15 | 25.51 | 25.16 | 25.52 |
| 26 | 26.00 | 26.35 | 25.94 | 26.28 | 26.18 | 26.54 | 26.19 | 26.56 |
| 27 | 27.00 | 27.35 | 26.91 | 27.26 | 27.20 | 27.56 | 27.22 | 27.58 |
| 28 | 28.00 | 28.34 | 27.89 | 28.23 | 28.22 | 28.57 | 28.24 | 28.58 |
| 29 | 29.00 | 29.32 | 28.86 | 29.18 | 29.24 | 29.56 | 29.25 | 29.57 |
| 30 | 30.00 | 30.29 | 29.84 | 30.13 | 30.26 | 30.55 | 30.27 | 30.56 |
| 31 | 31.00 | 31.26 | 30.82 | 31.08 | 31.28 | 31.53 | 31.28 | 31.53 |
| 32 | 32.00 | 32.22 | 31.81 | 32.04 | 32.28 | 32.49 | 32.27 | 32.48 |
| 33 | 33.00 | 33.18 | 32.83 | 33.02 | 33.26 | 33.41 | 33.24 | 33.40 |
| 34 | 34.00 | 34.13 | 33.87 | 34.02 | 34.19 | 34.30 | 34.18 | 34.29 |
| 35 | 35.00 | 35.07 | 34.94 | 35.02 | 35.10 | 35.16 | 35.09 | 35.15 |
| 36 | 36 | 36 | 36 | 36 | 36 | 36 | 36 | 36 |

Observed score

| | | | | | | | | |
|---|---|---|---|---|---|---|---|---|
| n | 83 | 70 | 87 | 86 | 87 | 75 | 80 | 62 |
| Mean | 27.30 | 26.67 | 27.05 | 25.72 | 26.40 | 23.87 | 26.63 | 23.10 |
| s.d. | 5.54 | 4.43 | 5.30 | 5.41 | 5.27 | 5.68 | 5.31 | 4.77 |
| s.e. | 0.61 | 0.53 | 0.57 | 0.58 | 0.56 | 0.66 | 0.59 | 0.61 |

Equated score

| | | | | | | | | |
|---|---|---|---|---|---|---|---|---|
| Mean | 27.30 | 26.95 | 26.96 | 25.90 | 26.55 | 24.17 | 26.75 | 23.38 |
| s.d. | 5.54 | 4.43 | 5.24 | 5.40 | 5.39 | 5.91 | 5.45 | 5.04 |
| s.e. | 0.61 | 0.53 | 0.56 | 0.58 | 0.58 | 0.68 | 0.61 | 0.64 |

Test bias

| | | | | | | | | |
|---|---|---|---|---|---|---|---|---|
| Bias | 0.00 | -0.28 | 0.09 | -0.18 | -0.14 | -0.30 | -0.13 | -0.28 |
| stand. | 0.00 | -0.06 | 0.02 | -0.03 | -0.03 | -0.05 | -0.02 | -0.06 |

**Model 4 (n=825)**

Test of fit to the GLLRM with DIF DM and IN and locally dependent items AB, BJ, EF, DH and IJ shown in Figure DD.

<u>Confirmatory test for presence of local dependence in model</u>

Standardized gamma coefficients

```
AB: Kid1 & Kid2         lr = 114.34 df = 16 p = 0.0000 Gamma =  0.41
BJ: Kid2 & Kid10        lr =  42.23 df = 16 p = 0.0004 Gamma =  0.14
DH: Kid4 & Kid8         lr =  93.26 df = 16 p = 0.0000 Gamma =  0.34
EF: Kid5 & Kid6         lr =  61.04 df = 16 p = 0.0000 Gamma =  0.24
IJ: Kid9 & Kid10        lr = 125.20 df = 16 p = 0.0000 Gamma =  0.36
```

<u>Confirmatory test for presence of DIF in model</u>

Standardized gamma coefficients

```
DK: Kid4 & Language     lr =  14.22 df =  4 p = 0.0066 gamma = -0.01
DM: Kid4 & Sex          lr =  10.66 df =  4 p = 0.0307 gamma = -0.18
EM: Kid5 & Sex          lr =  21.05 df =  4 p = 0.0003 gamma = -0.29
FK: Kid6 & Language     lr =  18.65 df =  4 p = 0.0009 gamma = -0.05
IM: Kid9 & Sex          lr =  13.72 df =  4 p = 0.0083 gamma =  0.25
```

<u>Summary of global test results (homogenity across scoregroups and invariance across exogenous variables)</u>

Delta will be reported if estimation did not converge

| | CLR | df | p |
|---|---|---|---|
| scoregroups | 160.7 | 130 | 0.035 |
| K: Language | 154.7 | 114 | 0.007 |
| L: School | 149.3 | 130 | 0.119 |
| M: Sex | 124.9 | 106 | 0.101 |
| N: Grade | 453.4 | 390 | 0.015 |

Critical levels adjusted by the Benjamini-Hochberg procedure:

```
 FDR = 0.05          reject if p<=  0.0500
 FDR = 0.01          reject if p<=  0.0020
 FDR = 0.001         reject if p<=  0.0002
```

<u>Tests of item fit</u>

Conditional outfits and infits

| Item | | Outfit observed | sd | p | Infit observed | sd | p |
|---|---|---|---|---|---|---|---|
| A - | Kid1 | 0.856 | 0.058 | 0.01232 | 0.883 | 0.053 | 0.02689 |
| B - | Kid2 | 1.008 | 0.049 | 0.87542 | 1.008 | 0.051 | 0.86999 |
| D - | Kid4 | 1.268 | 0.057 | 0.00000*** | 1.128 | 0.056 | 0.02172 |
| E - | Kid5 | 1.038 | 0.048 | 0.43668 | 1.036 | 0.050 | 0.47056 |
| F - | Kid6 | 1.076 | 0.051 | 0.13795 | 1.056 | 0.052 | 0.27780 |
| G - | Kid7 | 0.917 | 0.079 | 0.29546 | 0.968 | 0.071 | 0.64898 |
| H - | Kid8 | 0.988 | 0.066 | 0.85000 | 0.941 | 0.061 | 0.32882 |
| I - | Kid9 | 1.062 | 0.054 | 0.25103 | 1.035 | 0.055 | 0.52691 |

| | | | | | | | |
|---|---|---|---|---|---|---|---|
| J - | Kid10 | 0.992 | 0.054 | 0.87739 | 0.966 | 0.054 | 0.53219 |

Item restscore association

| Item | | Item-restscore gamma observed | expected | sd | p |
|---|---|---|---|---|---|
| A - | Kid1 | 0.629 | 0.559 | 0.025 | 0.00506* |
| B - | Kid2 | 0.580 | 0.575 | 0.025 | 0.85032 |
| D - | Kid4 | 0.486 | 0.513 | 0.027 | 0.31225 |
| E - | Kid5 | 0.497 | 0.509 | 0.026 | 0.65808 |
| F - | Kid6 | 0.485 | 0.504 | 0.027 | 0.47970 |
| G - | Kid7 | 0.484 | 0.419 | 0.035 | 0.06265 |
| H - | Kid8 | 0.559 | 0.508 | 0.030 | 0.08957 |
| I - | Kid9 | 0.493 | 0.513 | 0.028 | 0.47952 |
| J - | Kid10 | 0.584 | 0.564 | 0.026 | 0.45708 |

Critical levels adjusted by the Benjamini-Hochberg procedure:
* < 5 % FDR, ** < 1 % FDR, *** = FDR < 0.1 % FDR

Component-restscore gamma coefficients

| Component | Gamma observed | expected | sd | p |
|---|---|---|---|---|
| ABIJ | 0.548 | 0.526 | 0.021 | 0.3003 |
| DH | 0.487 | 0.491 | 0.024 | 0.8869 |
| EF | 0.479 | 0.499 | 0.023 | 0.3855 |

Benjamini-Hochberg limits for all outfits, infits and gamma coefficients

FDR = 5 %. Limit = 0.00167
FDR = 1 %. Limit = 0.00033

## Effect of DIF

*Item scores have been recoded for analysis (1-5 → 0-4), and thus the sum scale score range was changed from 9-45 to 0-36.*

DIF sources:
K - Language: 1 = Danish 2 = Other
M - Sex: 1 = Boy 2 = Girl

| score | KM 11 | KM 21 | KM 12 | KM 22 |
|---|---|---|---|---|
| 0 | 0 | 0 | 0 | 0 |
| 1 | 1.00 | 1.81 | 0.88 | 1.61 |
| 2 | 2.00 | 2.94 | 1.71 | 2.56 |
| 3 | 3.00 | 3.96 | 2.59 | 3.44 |
| 4 | 4.00 | 4.95 | 3.51 | 4.33 |
| 5 | 5.00 | 5.94 | 4.45 | 5.26 |
| 6 | 6.00 | 6.93 | 5.42 | 6.23 |
| 7 | 7.00 | 7.93 | 6.43 | 7.25 |
| 8 | 8.00 | 8.95 | 7.47 | 8.32 |
| 9 | 9.00 | 9.99 | 8.53 | 9.44 |
| 10 | 10.00 | 11.04 | 9.62 | 10.60 |

| | | | | |
|---|---|---|---|---|
| 11 | 11.00 | 12.12 | 10.72 | 11.78 |
| 12 | 12.00 | 13.20 | 11.83 | 12.96 |
| 13 | 13.00 | 14.27 | 12.93 | 14.14 |
| 14 | 14.00 | 15.32 | 14.02 | 15.27 |
| 15 | 15.00 | 16.34 | 15.09 | 16.35 |
| 16 | 16.00 | 17.31 | 16.13 | 17.36 |
| 17 | 17.00 | 18.23 | 17.14 | 18.31 |
| 18 | 18.00 | 19.12 | 18.13 | 19.20 |
| 19 | 19.00 | 19.97 | 19.09 | 20.04 |
| 20 | 20.00 | 20.81 | 20.04 | 20.86 |
| 21 | 21.00 | 21.64 | 20.98 | 21.66 |
| 22 | 22.00 | 22.48 | 21.93 | 22.48 |
| 23 | 23.00 | 23.35 | 22.88 | 23.30 |
| 24 | 24.00 | 24.23 | 23.85 | 24.16 |
| 25 | 25.00 | 25.15 | 24.84 | 25.05 |
| 26 | 26.00 | 26.08 | 25.85 | 25.98 |
| 27 | 27.00 | 27.03 | 26.86 | 26.93 |
| 28 | 28.00 | 27.99 | 27.88 | 27.90 |
| 29 | 29.00 | 28.96 | 28.91 | 28.89 |
| 30 | 30.00 | 29.94 | 29.95 | 29.90 |
| 31 | 31.00 | 30.92 | 30.98 | 30.91 |
| 32 | 32.00 | 31.91 | 32.02 | 31.92 |
| 33 | 33.00 | 32.90 | 33.05 | 32.94 |
| 34 | 34.00 | 33.90 | 34.07 | 33.96 |
| 35 | 35.00 | 34.92 | 35.06 | 34.98 |
| 36 | 36 | 36 | 36 | 36 |

Observed score

| | | | | |
|---|---|---|---|---|
| n | 315 | 114 | 306 | 100 |
| Mean | 27.70 | 27.39 | 25.44 | 23.86 |
| s.d. | 4.68 | 5.55 | 5.27 | 6.49 |
| s.e. | 0.26 | 0.52 | 0.30 | 0.65 |

Equated score

| | | | | |
|---|---|---|---|---|
| Mean | 27.70 | 27.56 | 25.38 | 24.20 |
| s.d. | 4.68 | 5.25 | 5.26 | 6.10 |
| s.e. | 0.26 | 0.49 | 0.30 | 0.61 |

Test bias

| | | | | |
|---|---|---|---|---|
| Bias | 0.00 | -0.16 | 0.06 | -0.34 |
| stand. | 0.00 | -0.03 | 0.01 | -0.06 |

**Model 5 (n=1050)**
Test of fit to the GLLRM with DIF AM, DM, EM, HM and IN and locally dependent items AB, AD, EF, DH and IJ shown in Figure EE.

Confirmatory test for presence of local dependence in model

```
Standardized gamma coefficients

AB: Kid1 & Kid2         lr = 133.21 df = 16 p = 0.0000 Gamma =  0.35
AD: Kid1 & Kid4         lr =  47.24 df = 16 p = 0.0001 Gamma =  0.10
DH: Kid4 & Kid8         lr =  68.00 df = 16 p = 0.0000 Gamma =  0.26
EF: Kid5 & Kid6         lr = 125.22 df = 16 p = 0.0000 Gamma =  0.37
IJ: Kid9 & Kid10        lr = 149.88 df = 16 p = 0.0000 Gamma =  0.41
```

Confirmatory test for presence of DIF in model

```
Standardized gamma coefficients

AM: Kid1 & Sex          lr =  19.67 df =  4 p = 0.0006 gamma = -0.28
DM: Kid4 & Sex          lr =  51.32 df =  4 p = 0.0000 gamma = -0.42
EM: Kid5 & Sex          lr =  12.30 df =  4 p = 0.0153 gamma = -0.08
HM: Kid8 & Sex          lr =  20.43 df =  4 p = 0.0004 gamma = -0.18
IN: Kid9 & Grade        lr =  40.15 df = 12 p = 0.0001 gamma = -0.18
```

Summary of global test results (homogenity across scoregroups and invariance across exogenous variables)

```
Delta will be reported if estimation did not converge

             CLR   df    p
----------------------------
scoregroups  127.1 139 0.757
K: Language  171.8 139 0.031
L:   School  129.2 139 0.713
M:      Sex  118.8 107 0.206
N:    Grade  416.9 369 0.043

Critical levels adjusted by the Benjamini-Hochberg procedure:

 FDR = 0.05          reject if p<=  0.0100
 FDR = 0.01          reject if p<=  0.0020
 FDR = 0.001         reject if p<=  0.0002
```

Tests of item fit

```
Conditional outfits and infits

               Outfit                              Infit
Item          observed    sd       p        observed     sd       p
-----------------------------------------------------------------------
A -     Kid1    0.852    0.054  0.00672*       0.884    0.048  0.01588
B -     Kid2    1.017    0.044  0.69981        0.996    0.046  0.92239
D -     Kid4    1.187    0.052  0.00029**      1.128    0.050  0.01100
E -     Kid5    1.043    0.043  0.32469        1.051    0.044  0.25331
F -     Kid6    0.967    0.046  0.46809        0.989    0.046  0.81613
G -     Kid7    1.081    0.069  0.24093        1.034    0.062  0.58593
```

```
H -      Kid8     0.898     0.057  0.07234        0.944     0.055  0.31040
I -      Kid9     1.044     0.048  0.35955        1.035     0.049  0.46850
J -     Kid10     0.987     0.046  0.78038        0.976     0.047  0.61779
-----------------------------------------------------------------------

Item restscore association

                   Item-restscore gamma
Item            observed expected    sd       p
------------------------------------------------
A -      Kid1     0.631     0.570     0.022  0.00507*
B -      Kid2     0.538     0.534     0.023  0.87136
D -      Kid4     0.467     0.520     0.024  0.02775
E -      Kid5     0.503     0.526     0.023  0.32046
F -      Kid6     0.533     0.523     0.023  0.66923
G -      Kid7     0.467     0.408     0.030  0.05298
H -      Kid8     0.521     0.488     0.027  0.23171
I -      Kid9     0.500     0.519     0.024  0.43692
J -     Kid10     0.537     0.519     0.025  0.47160
------------------------------------------------

Critical levels adjusted by the Benjamini-Hochberg procedure:
    * < 5 % FDR, ** < 1 % FDR, *** = FDR < 0.1 % FDR


Component-restscore gamma coefficients

                    Gamma
Component       observed expected    sd       p
------------------------------------------------
  ABDH            0.510     0.519     0.019   0.6481
    EF            0.486     0.492     0.021   0.7835
    IJ            0.480     0.478     0.022   0.9382


Benjamini-Hochberg limits for all outfits, infits and gamma coefficients

FDR = 5 %. Limit =  0.00167
FDR = 1 %. Limit =  0.00033
```

<u>Effect of DIF</u>

*Item scores have been recoded for analysis (1-5 → 0-4), and thus the sum scale score range was changed from 9-45 to 0-36.*

```
DIF sources:
M -      Sex: 1 = Boy  2 = Girl
N -    Grade: 1 = 5   2 = 6   3 = 7   4 = 8

            MN      MN      MN      MN      MN      MN      MN      MN
score       11      21      12      22      13      23      14      24
---------------------------------------------------------------
    0        0       0       0       0       0       0       0       0
    1     1.00    1.11    1.23    1.11    1.23    1.11    1.24    1.11
    2     2.00    1.60    1.93    1.57    1.92    1.56    1.91    1.56
    3     3.00    2.46    2.89    2.38    2.86    2.36    2.79    2.33
    4     4.00    3.44    3.87    3.32    3.84    3.29    3.68    3.18
    5     5.00    4.47    4.88    4.34    4.84    4.30    4.60    4.09
    6     6.00    5.54    5.90    5.42    5.87    5.38    5.55    5.08
    7     7.00    6.64    6.94    6.56    6.91    6.52    6.54    6.13
```

| | | | | | | | | |
|---|---|---|---|---|---|---|---|---|
| 8 | 8.00 | 7.77 | 7.99 | 7.74 | 7.96 | 7.71 | 7.55 | 7.26 |
| 9 | 9.00 | 8.92 | 9.04 | 8.96 | 9.02 | 8.94 | 8.60 | 8.45 |
| 10 | 10.00 | 10.09 | 10.09 | 10.20 | 10.09 | 10.20 | 9.65 | 9.69 |
| 11 | 11.00 | 11.26 | 11.14 | 11.45 | 11.15 | 11.46 | 10.72 | 10.96 |
| 12 | 12.00 | 12.43 | 12.19 | 12.67 | 12.21 | 12.70 | 11.80 | 12.23 |
| 13 | 13.00 | 13.59 | 13.23 | 13.88 | 13.26 | 13.93 | 12.87 | 13.49 |
| 14 | 14.00 | 14.72 | 14.27 | 15.06 | 14.32 | 15.12 | 13.95 | 14.73 |
| 15 | 15.00 | 15.84 | 15.30 | 16.20 | 15.36 | 16.28 | 15.02 | 15.93 |
| 16 | 16.00 | 16.93 | 16.33 | 17.32 | 16.41 | 17.41 | 16.09 | 17.10 |
| 17 | 17.00 | 18.01 | 17.36 | 18.41 | 17.45 | 18.53 | 17.15 | 18.24 |
| 18 | 18.00 | 19.07 | 18.38 | 19.49 | 18.48 | 19.62 | 18.21 | 19.36 |
| 19 | 19.00 | 20.13 | 19.40 | 20.56 | 19.52 | 20.70 | 19.27 | 20.47 |
| 20 | 20.00 | 21.18 | 20.41 | 21.62 | 20.54 | 21.78 | 20.32 | 21.56 |
| 21 | 21.00 | 22.23 | 21.42 | 22.67 | 21.57 | 22.84 | 21.36 | 22.64 |
| 22 | 22.00 | 23.27 | 22.42 | 23.71 | 22.58 | 23.89 | 22.39 | 23.72 |
| 23 | 23.00 | 24.31 | 23.42 | 24.74 | 23.60 | 24.94 | 23.42 | 24.77 |
| 24 | 24.00 | 25.34 | 24.42 | 25.75 | 24.61 | 25.96 | 24.44 | 25.81 |
| 25 | 25.00 | 26.35 | 25.41 | 26.75 | 25.61 | 26.96 | 25.46 | 26.82 |
| 26 | 26.00 | 27.35 | 26.40 | 27.72 | 26.61 | 27.93 | 26.46 | 27.80 |
| 27 | 27.00 | 28.32 | 27.38 | 28.66 | 27.60 | 28.87 | 27.46 | 28.75 |
| 28 | 28.00 | 29.26 | 28.37 | 29.57 | 28.59 | 29.78 | 28.46 | 29.66 |
| 29 | 29.00 | 30.17 | 29.34 | 30.44 | 29.57 | 30.65 | 29.44 | 30.53 |
| 30 | 30.00 | 31.05 | 30.31 | 31.29 | 30.55 | 31.49 | 30.42 | 31.37 |
| 31 | 31.00 | 31.90 | 31.28 | 32.10 | 31.51 | 32.29 | 31.38 | 32.18 |
| 32 | 32.00 | 32.73 | 32.24 | 32.90 | 32.47 | 33.08 | 32.33 | 32.96 |
| 33 | 33.00 | 33.54 | 33.20 | 33.67 | 33.40 | 33.84 | 33.27 | 33.73 |
| 34 | 34.00 | 34.35 | 34.14 | 34.44 | 34.32 | 34.58 | 34.19 | 34.48 |
| 35 | 35.00 | 35.16 | 35.08 | 35.22 | 35.20 | 35.31 | 35.10 | 35.24 |
| 36 | 36 | 36 | 36 | 36 | 36 | 36 | 36 | 36 |

Observed score

| | | | | | | | | |
|---|---|---|---|---|---|---|---|---|
| n | 134 | 139 | 137 | 135 | 123 | 131 | 120 | 131 |
| Mean | 27.20 | 26.14 | 27.15 | 25.94 | 26.78 | 25.35 | 26.76 | 23.99 |
| s.d. | 5.35 | 4.74 | 4.89 | 5.26 | 4.97 | 5.22 | 5.06 | 5.28 |
| s.e. | 0.46 | 0.40 | 0.42 | 0.45 | 0.45 | 0.46 | 0.46 | 0.46 |

Equated score

| | | | | | | | | |
|---|---|---|---|---|---|---|---|---|
| Mean | 27.20 | 27.26 | 27.48 | 27.34 | 27.31 | 26.97 | 27.12 | 25.46 |
| s.d. | 5.35 | 4.63 | 4.84 | 5.07 | 4.95 | 5.12 | 5.10 | 5.27 |
| s.e. | 0.46 | 0.39 | 0.41 | 0.44 | 0.45 | 0.45 | 0.47 | 0.46 |

Test bias

| | | | | | | | | |
|---|---|---|---|---|---|---|---|---|
| Bias | 0.00 | -1.12 | -0.33 | -1.40 | -0.53 | -1.62 | -0.37 | -1.47 |
| stand. | 0.00 | -0.24 | -0.07 | -0.28 | -0.11 | -0.32 | -0.07 | -0.28 |

**Non-fitting Model 6 (n=1651)**

Test of fit to the GLLRM with DIF DM, EM, GM, EK, BN and IN and locally dependent items AB, AD, AH, EF, DH, FH and IJ shown in Figure FF.

Confirmatory test for presence of local dependence in model

```
Standardized gamma coefficients will be reported.

AB: Kid1 & Kid2        lr = 252.08 df = 16 p = 0.0000 Gamma =  0.35
AD: Kid1 & Kid4        lr =  57.17 df = 16 p = 0.0000 Gamma =  0.15
AH: Kid1 & Kid8        lr =  69.85 df = 16 p = 0.0000 Gamma =  0.20
DH: Kid4 & Kid8        lr = 138.56 df = 16 p = 0.0000 Gamma =  0.30
EF: Kid5 & Kid6        lr = 166.87 df = 16 p = 0.0000 Gamma =  0.25
FH: Kid6 & Kid8        lr =  55.45 df = 16 p = 0.0000 Gamma =  0.08
IJ: Kid9 & Kid10       lr = 221.97 df = 16 p = 0.0000 Gamma =  0.46
```

Confirmatory test for presence of DIF in model

```
Standardized gamma coefficients will be reported.

BN: Kid2 & Grade       lr =  32.57 df = 12 p = 0.0011 gamma = -0.15
DM: Kid4 & Sex         lr =  31.24 df =  4 p = 0.0000 gamma = -0.27
EK: Kid5 & Language    lr =  28.77 df =  4 p = 0.0000 gamma =  0.15
EM: Kid5 & Sex         lr =  26.41 df =  4 p = 0.0000 gamma = -0.17
GM: Kid7 & Sex         lr =  24.21 df =  4 p = 0.0001 gamma =  0.14
IN: Kid9 & Grade       lr =  37.50 df = 12 p = 0.0002 gamma = -0.18
```

Summary of global test results (homogenity across scoregroups and invariance across exogenous variables)

Delta will be reported if estimation did not converge

| | CLR | df | p | delta |
|---|---|---|---|---|
| scoregroups | 279.5 | 184 | 0.000 | |
| K: Language | 256.7 | 176 | 0.000 | |
| L: School | 238.4 | 184 | 0.004 | |
| M: Sex | 170.5 | 160 | 0.271 | |
| N: Grade | 1077.1 | 456 | 0.000 | 108.155 |

Critical levels adjusted by the Benjamini-Hochberg procedure:

```
 FDR = 0.05          reject if p<=  0.0500
 FDR = 0.01          reject if p<=  0.0100
 FDR = 0.001         reject if p<=  0.0010
```

Tests of item fit

Conditional outfits and infits

| Item | | Outfit observed | sd | p | Infit observed | sd | p |
|---|---|---|---|---|---|---|---|
| A - | Kid1 | 0.919 | 0.044 | 0.06582 | 0.913 | 0.040 | 0.02932 |
| B - | Kid2 | 0.993 | 0.036 | 0.84707 | 0.981 | 0.037 | 0.60443 |
| D - | Kid4 | 1.147 | 0.041 | 0.00035** | 1.110 | 0.040 | 0.00561 |
| E - | Kid5 | 1.014 | 0.036 | 0.69247 | 1.007 | 0.036 | 0.83413 |
| F - | Kid6 | 0.993 | 0.041 | 0.85513 | 0.984 | 0.040 | 0.68207 |
| G - | Kid7 | 0.978 | 0.058 | 0.70504 | 0.974 | 0.051 | 0.61797 |
| H - | Kid8 | 0.956 | 0.058 | 0.45315 | 0.995 | 0.047 | 0.90888 |
| I - | Kid9 | 1.084 | 0.036 | 0.01872 | 1.079 | 0.037 | 0.03179 |
| J - | Kid10 | 0.948 | 0.037 | 0.16024 | 0.962 | 0.038 | 0.31385 |

```
--------------------------------------------------------------------------

Item restscore association

                   Item-restscore gamma
Item           observed expected    sd      p
-------------------------------------------------
A -      Kid1    0.627    0.580    0.017  0.00642*
B -      Kid2    0.527    0.514    0.019  0.51401
D -      Kid4    0.482    0.512    0.019  0.12259
E -      Kid5    0.483    0.472    0.019  0.59664
F -      Kid6    0.512    0.489    0.020  0.25563
G -      Kid7    0.431    0.366    0.025  0.00935*
H -      Kid8    0.555    0.553    0.021  0.92537
I -      Kid9    0.420    0.483    0.020  0.00124*
J -     Kid10    0.515    0.490    0.021  0.22980
-------------------------------------------------

Critical levels adjusted by the Benjamini-Hochberg procedure:
    * < 5 % FDR, ** < 1 % FDR, *** = FDR < 0.1 % FDR

Component-restscore gamma coefficients

                    Gamma
Component      observed expected    sd      p
-------------------------------------------------
ABDEFH           0.437    0.460    0.017   0.1755
    IJ           0.421    0.442    0.018   0.2384

Benjamini-Hochberg limits for all outfits, infits and gamma coefficients

FDR = 5 %. Limit =  0.00690
FDR = 1 %. Limit =  0.00034
```

<u>Confirmatory test of unidimensionality across the sub-component ABDEFH and the remaining three items; GIJ</u>

```
Observed and expected subscore correlation

Expected Gamma =   0.460  s.e. =  0.0168
Observed Gamma =   0.437    p  =  0.1755  (Two-sided)


Monte Carlo estimate of p-values. Sample size = 1000

Gamma:                     p(below) =  0.036
```
